# Lost genome segments associate with trait diversity during rice domestication

**DOI:** 10.1186/s12915-023-01512-6

**Published:** 2023-02-01

**Authors:** Xiaoming Zheng, Limei Zhong, Hongbo Pang, Siyu Wen, Fei Li, Danjing Lou, Jinyue Ge, Weiya Fan, Tianyi Wang, Zhenyun Han, Weihua Qiao, Xiaowu Pan, Yebao Zhu, Jilin Wang, Cuifeng Tang, Xinhua Wang, Jing Zhang, Zhijian Xu, Sung Ryul Kim, Ajay Kohli, Guoyou Ye, Kenneth M. Olsen, Wei Fang, Qingwen Yang

**Affiliations:** 1 National Key Facility for Crop Gene Resources and Genetic Improvement, Institute of Crop Sciences, Chinese Academy of Agricultural Sciences, Beijing, 100081 China; 2 International Rice Research Institute, DAPO box 7777, Metro Manila, the Philippines; 3 Sanya National Research Institute of Breeding in Hainan, Chinese Academy of Agricultural Sciences, Beijing, China; 4 College of life science, Nanchang University, Nanchang, China; 5 College of Life Science, Shenyang Normal University, Shenyang, China; 6 Smartgenomics Technology Institute, Tianjin, China; 7 Rice Research Institute, Hunan Academy of Agricultural Sciences, Changsha, China; 8 Rice Research Institute, Fujian Academy of Agricultural Sciences, Fuzhou, China; 9 Rice Research Institute, Jiangxi Academy of Agricultural Sciences, Nanchang, China; 10 Biotechnology and Germplasm Resources Institute, Yunnan Academy of Agricultural Sciences, Kunming, China; 11 Institute of Food Crops, Hainan Academy of Agricultural Sciences, Haikou, China; 12 Rice Research Institute, Guangdong Academy of Agricultural Sciences, Guangzhou, China; 13 Guangdong Provincial Key Laboratory of New Technology in Rice Breeding, Guangzhou, China; 14 Rice Research Institute, Guangxi Academy of Agricultural Sciences, Nanning, China; 15 Crop Biotech Institute & Department of Genetic Engineering, Kyung Hee University, Yongin, 446-701 Republic of Korea; 16 Biology Department, Washington University, Campus Box 1137, St. Louis, MO 63130 USA

**Keywords:** Structure variation, *Oryza*, Domestication, Phenotype, Association

## Abstract

**Background:**

DNA mutations of diverse types provide the raw material required for phenotypic variation and evolution. In the case of crop species, previous research aimed to elucidate the changing patterns of repetitive sequences, single-nucleotide polymorphisms (SNPs), and small InDels during domestication to explain morphological evolution and adaptation to different environments. Additionally, structural variations (SVs) encompassing larger stretches of DNA are more likely to alter gene expression levels leading to phenotypic variation affecting plant phenotypes and stress resistance. Previous studies on SVs in rice were hampered by reliance on short-read sequencing limiting the quantity and quality of SV identification, while SV data are currently only available for cultivated rice, with wild rice largely uncharacterized. Here, we generated two genome assemblies for *O. rufipogon* using long-read sequencing and provide insights on the evolutionary pattern and effect of SVs on morphological traits during rice domestication.

**Results:**

In this study, we identified 318,589 SVs in cultivated and wild rice populations through a comprehensive analysis of 13 high-quality rice genomes and found that wild rice genomes contain 49% of unique SVs and an average of 1.76% of genes were lost during rice domestication. These SVs were further genotyped for 649 rice accessions, their evolutionary pattern during rice domestication and potential association with the diversity of important agronomic traits were examined. Genome-wide association studies between these SVs and nine agronomic traits identified 413 candidate causal variants, which together affect 361 genes. An 824-bp deletion in japonica rice, which encodes a serine carboxypeptidase family protein, is shown to be associated with grain length.

**Conclusions:**

We provide relatively accurate and complete SV datasets for cultivated and wild rice accessions, especially in TE-rich regions, by comparing long-read sequencing data for 13 representative varieties. The integrated rice SV map and the identified candidate genes and variants represent valuable resources for future genomic research and breeding in rice.

**Supplementary Information:**

The online version contains supplementary material available at 10.1186/s12915-023-01512-6.

## Introduction

Structural variations (SVs), which affect plant phenotypes and biotic and abiotic resistance [1–3], have been shown to play an essential role in plant evolution and agricultural research. SVs can result in large-scale perturbations of *cis*-regulatory regions compared to single-nucleotide polymorphisms (SNPs) [4]; therefore, SVs are more prone to alter gene expression levels that ultimately lead to phenotype variations [5, 6]. However, the pervasiveness and functional impacts of SVs in most plant genomes remain elusive [7]. Key unanswered questions include the following: What is the proportion of SVs compared with that of SNPs? What percentage of genes across the genome are influenced by SVs? How does the SV diversity change during domestication? The answers to the above questions are required to evaluate the force that shapes the evolutionary fate of SVs, to access the association between SVs and phenotypes, and to adopt them as an approach to understand critical processes such as speciation, domestication, and adaptation.

The identification of SVs using short sequencing reads alone is especially challenging and unreliable, as reliance on this approach would result in a great number of undetected SVs and their phenotypic and molecular influences would be obscured [8, 9]. An SV may span a large portion of a read or even be longer than the read; thus, it is obviously that the low detection rate of long insertions caused by tandem duplications in the identification of SVs using short sequencing reads, and multiple SVs can sometimes overlap or be nested [10]. Long-read sequencing is superior for SV identification because it can span repetitive or other problematic regions and shows the potential to improve the mapping quality, capturing larger SVs more precisely relative to short reads alone [7, 11].

Asian cultivated rice (*Oryza sativa* L.) is one of the most essential staple foods for half the world, contributing about 20% of the human dietary calories [12]. To attain the critical food demand of the world’s growing population and to cope with the global climate change in future, it is urgent for rice breeders to develop new rice resources harboring higher and more stable yields as well as create rice varieties that would be able to adapt to various environmental conditions. Asian cultivated rice has been revealed to be domesticated from an immediate wild ancestor, *O. rufipogon*, thousands of years ago [12, 13]. Compared to *O. rufipogon*, cultivated rice has undergone considerable physiological and phenotypic alterations and a substantial loss of genetic diversity through artificial selection and successive bottlenecks for agronomically important traits [14, 15]. Additionally, *O*. *rufipogon* is distributed across broad geographical ranges and natural habitats and can tolerate and adapt to various abiotic and biotic stress conditions [16]. Thus, the wild relatives of rice constitute a crucial pool for improving rice germplasm, which is of great significance to ensure the rice production and food security worldwide.

It has been demonstrated that large population samples are the foundation to investigate SVs in Asian rice. For instance, Fuentes et al. [4] identified an SV set from 3000 cultivated rice varieties based on short-read sequencing with an average coverage of 14×. However, the coverage of some rice accessions was too low to yield reliable SV results [11]. Nevertheless, they managed to apply ten different programs for SV detection. Carpentier et al. [17] employed the same dataset to detect SVs generated by retrotransposon insertions and revealed that mobile element insertions were present in 80% of SVs. This result indicated that either recent transposable element activities in domestication or selection against mobile element insertions reached a high frequency [18–20]. So far, the population frequency of single SV events has only been analyzed in grape, peach, and rice, and these studies have relied on short-read sequences. Knowledge about the population demographic role of SVs in the formation or domestication of rice phenotypic diversity is scarce. In the present study, (1) we generated two genome assemblies for *O. rufipogon* by integrating Nanopore long-read sequencing and Hi-C technology; (2) we identified SVs among wild and cultivated rice genomes combining these two genomes and previously published 13 genome assemblies constructed with both high-coverage PacBio and Illumina sequencing data; (3) we further genotyped these SVs in an extended population comprised by 169 wild and 480 cultivated rice accessions, and identified 413 SVs associated with nine important agriculture traits.

## Results

### Genome assembly and annotation of wild rice

**Fig. 1:**
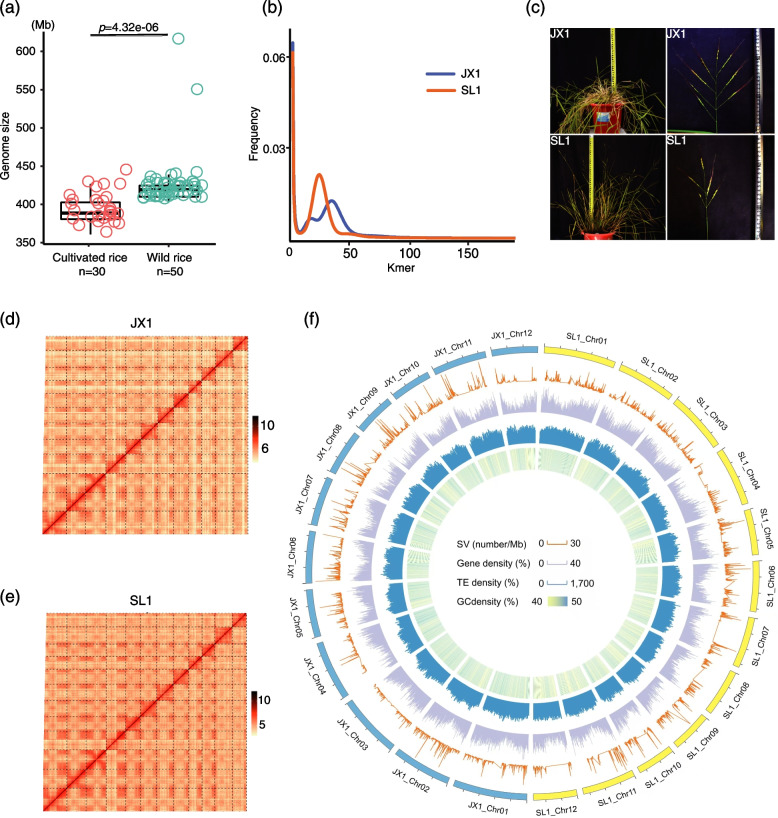
Genomic landscape of the JX1 and SL1 genomes. **a** Genome size of cultivated and wild rice based on genome survey results. **b** Distribution of k-mers for JX1 and SL1. **c** Phenotype of the plant and panicle of JX1 and SL1. **d, e** Genome-wide chromosome heatmap of the Hi-C data for JX1 and SL1. **f** SV number distribution and gene, transposable element, and GC content densities in 100-kb sliding windows

To estimate the size and complexity of wild and cultivated rice genomes, we performed genome survey analysis with Illumina sequencing data for 80 samples, including 50 globally distributed wild rice accessions and 30 cultivated rice varieties representing broad genetic variations (Additional file 2: Table S1). We found that the genome size of wild rice (average 424 Mb) was significantly larger than that of cultivated rice (average 393 Mb) (*t*-test, *p* = 4.32×10^-6^; Fig. 1a). It is possible that some genomic fragments were lost during domestication, and the supplement of the wild rice genome will help us understand the domestication process of cultivated rice. After integrating information on heterozygosity (Additional file 2: Table S1; Fig. 1b), geographical location, morphological traits (Fig. 1c), and phylogenetic relationships of cultivated rice (Additional file 1: Fig. S1), we selected the rice accession JX1 (with low heterozygosity and genetic similarity to *O. sativa* ssp. *japonica*) from Jiangxi Province of China and SL1 (with a high heterozygosity rate of 0.9% and genetic similarity to *O. sativa* ssp. *indica*) from Sri Lanka for subsequent genome sequencing and assembly.

To assemble the JX1 and SL1 genomes, we employed a combination of long-read sequencing (Additional file 2: Table S2, S3), chromosome karyotyping (Additional file 1: Fig. S2), and Hi-C sequencing technologies (Additional file 2: Table S4). The final assemblies of JX1 and SL1 were 409.3 and 396.4 Mb in length, respectively, with 98.7% and 99.2% of sequences anchored to 12 chromosomes (Table 1). The quality of these genome assemblies of JX1 and SL1 were evaluated from four aspects. First, the corrected reads were evenly distributed along the chromosomes (Additional file 1: Fig. S3). Second, both the genome assemblies exhibited excellent collinearity with the Nipponbare genome (Additional file 1: Fig. S4). Third, the benchmarking universal single-copy orthologs (BUSCO) evaluation revealed that 98.1 and 96.4% of the 1440 single-copy Embryophyta genes were completely assembled in the JX1 and SL1 genomes (Additional file 2: Table S5). Fourth, the chromatin interaction matrix of the 12 chromosomes derived from Hi-C sequencing showed that the interactions were mainly present within proximity to one another within the same chromosome, with the strongest signal along the diagonal and no apparent inter-chromosomal hotspots (Additional file 1: Fig. S1d-f). These observations indicated the assembled JX1 and SL1 genomes are of high quality.

**Table 1:** Summary statistics for the JX1 and SL1 assembly and their genomic annotations

Categories	Genomic feature	JX1	SL1
Genome	Estimated size (Mb)	424.9	412.7
	GC content (%)	43%	44%
	Length of assembly (Mb)	409.3	396.4
	Contig N50 (bp)	5,825,388	2,665,012
	Contig N90 (bp)	1,095,364	669,592
	Largest contig (bp)	15,735,557	19,511,849
	Sequences anchored to chromosomes (%)	98.7%	99.2%
	Complete BUSCOs (%)	98.1%	96.4%
Genes	Genes/transcripts (#)	39,252/53,210	39,343/45,613
	Novel genes/transcripts (#)	3,178/33,568	2,467/31,234
	Fusion genes (#)	477	260
	Mean transcript length (bp)	2910.5	2834.3
	Mean coding sequence length (bp)	1045.9	1000.4
TEs	Total size (bp)	200,708,242	186,636,582
	Class I: Retrotransposon (bp)	115,342,918	103,171,575
	Class II: DNA Transposon (bp)	85,365,324	83,465,007

Approximately 49.96 and 48.07% of the JX1 and SL1 genomes were annotated as repetitive DNA, respectively (Table 1 and Additional file 2: Table S6). They were mainly composed of transposable elements (TEs), and long terminal repeat (LTR)-retrotransposons were the most abundant subclass, consistent with previously reported rice genomes [21, 22]. To predict the protein-coding genes, we integrated three strategies, de novo predication based on sequence characteristics, homology searching against other well-annotated plant genomes, and transcriptome characterization with the Iso-seq method on a PacBio Sequel platform (Additional file 2: Table S7, S8). We merged the results predicted by the above three methods and finally obtained 53,210 and 45,613 isoforms, corresponding to 39,252 and 39,343 genes for JX1 and SL1, respectively (Table 1; Additional file 2: Table S9). To identify novel genes in wild rice, we first constructed a collection of 338,151 annotation genes of nine cultivated rice genomes. Then we compared the annotated genes of wild rice accessions (W_CAS, JX1, and SL1) to the collection of annotation genes from all cultivated rice group, and 27 (W_CAS), 316 (JX1), and 691 (SL1) novel genes were found in three genomes, respectively. The coding sequences of these novel genes cannot find the best matches of each 8 bps to the cultivated genomic segment. These observations show that at most only 1.76% of genes are completely lost during rice domestication. In addition, we found that some gene sequences were aligned to the collection of 338,151 annotation genes of nine cultivated rice genomes, but the aligned sequences did not have a complete open reading frame (noORF) in cultivated rice genome. These noORF genes account for about 30% of the genome-wide genes (JX1: 29.30%, SL1:39.80%, and W_CAS: 33.46%). These noORF genes may be the source of phenotypic differences between wild and cultivated rice.

### Identification and characterization of SVs

**Fig. 2:**
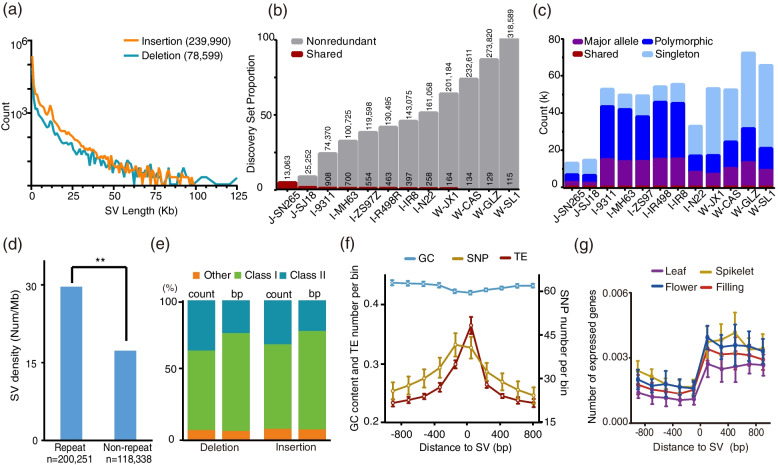
SV discovery. **a** Histograms illustrating frequencies of different sizes of SVs. **b** SVs from each sample were merged using a nonredundant strategy starting with J-SN265 and iteratively adding unique calls from additional samples. The growth rate of the nonredundant SVs declines as the number of samples increases. SVs shared among all samples are shown as red portions of each bar. **c** Stacked bar graphs showing the proportion of repeat types for all annotated insertions and deletions. Count, the proportion of individual repeat annotations; bp, the proportion of cumulative repeat sequence length; Other, other repeat types. **d** Structural variation density from repeat/non-repeat genomic regions in continuous 200-kb windows. Significance was tested by Fisher’s exact test; ***, *p* < 0.001. **e** Frequency for each variant type (insertion and deletion). Compared to deletions, a greater proportion of insertions were shared among all rice accessions. **f, g** Comparisons of the TE number (**f**), GC content (**f**), single-nucleotide mutation rate (**f**), and gene expression level (**g**) between SV and non-SV regions. Data are presented as means ± 95% CI

To discover SVs between wild and cultivated rice, we included an additional 11 published, high-quality, chromosome-scale rice genomes (Additional file 2: Table S10) in our study. These genomes included two *O. rufipogon* accessions (W-CAS and W-GLZ), two *O. sativa* ssp. *japonica* accessions (J-SJ18 and J-SN265), and six *O. sativa* ssp. *indica* accessions (I-9311, I-IR8, I-MH63, I-N22, I-R498, and I-ZS97). Then, we mapped all 12 genomes separately onto the Nipponbare genome and identified insertions and deletions of 50 bp or greater as SV targets. On average, we established 53,724 SVs with a size of 64 Mb per rice accession (Additional file 2: Table S11). Consistent with earlier observations [4, 7, 17], 87.3% of the identified SVs were novel. Our 13 samples combined yielded the most comprehensive sequence-resolved SV dataset involving cultivated and wild rice to date with a total of 318,589 nonredundant SVs, sized from 50 bp to 155.5 kb (Fig. 2a). Notably, a large number of the remaining SVs were now sequence-resolved for the first time; some randomly selected SVs are illustrated in Additional file 1: Fig. S5. Seven SVs with different sizes were amplified by PCR (Additional file 2: Table S12) and confirmed by Sanger sequencing.

The 318,589 SVs were comprised by 239,990 insertions and 78,599 deletions. The longest insertion and deletion was 99.5 kb and 155.5 kb in size, but most of the indels (>80%) are less than 1 kb and their number decreased as its size increased (Fig. 2a). As expected, the total number of SVs accumulated with the increasing individuals, and the accumulation rate of nonredundant SVs of *O. sativa* ssp. *japonica* (J-SJ18) vs. *O. sativa* ssp. *indica* (I-9311), *O. sativa* ssp. *japonica* (I-N22) vs. *O. rufipogon* (W-JX1), and all comparisons between wild rice accessions was obviously steeper than that of comparisons between cultivated rice accessions (Fig. 2b). Only 115 SVs were shared among all samples (Fig. 2b). These observations indicated that the variations are largely attributable to the divergence between the two cultivated rice subspecies and between cultivated and wild rice populations. The SVs were classified into four categories based on their frequency (shared, SVs occurring in all the rice accessions; major, SVs occurring in ≥ 50% of the rice accessions but not all; polymorphic, SVs occurring in more than one but < 50% of the rice accessions; and singleton, SVs occurring in only one rice accession) and found the polymorphic and singleton SVs together accounted for about 95% of all the SVs (Fig. 2c). The number of SVs in each of the 12 genomes was subsequently examined (Fig. 2c). The wild rice genomes had most SVs or similar SV numbers to the *indica* varieties, and the two *japonica* varieties harbored the least variations (Fig. 2c). A logical explanation for the observation is that the Nipponbare genome used as the reference here is a typical *japonica* variety. For the cultivated rice group (8 accessions), the increment of nonredundant SVs was initially steep; however, it declined with the addition of samples, indicating that a large number of shared variations are captured by the cultivated rice group. Moreover, we found that singletons mainly contributed to the SVs in all the four wild rice genomes, and the proportion of singletons was obviously greater in wild rice genomes than in cultivated rice genomes (Fig. 2c). As expected, the wild rice accessions exhibited the greatest genetic diversity, with each accession contributing 12.3% of the singleton SVs vs. 4.4% of cultivated rice accessions (Fig. 2c, Additional file 1: Fig. S6). These results suggested the abundant SVs present in *O. rufipogon* are providing a unique resource for rice breeding in the future.

### Genic and potential regulatory SVs

We did not detect significant differences in SV density for the subtelomeric (*p* = 0.0467, permutation; Additional file 1: Fig. S7a) and centromeric regions (*p* = 0.0268, permutation; Additional file 1: Fig. S7b). There was no greater subtelomeric bias observed in the chromosome long arms compared with the short arms (Additional file 1: Fig. S7c). However, SVs were nonrandomly distributed in the genome, with SV density generally elevated in regions rich in segmental duplications and repeats (Fig. 2d). The number of SVs that overlapped with repeat regions was 200,251, which was significantly larger than that within non-repeat regions (*p* = 0.006). Almost all the SV repeat bias was driven by tandem repeat sequences, with no elevation observed in SVs originating from retrotransposition (Fig. 2e; e.g., LTRs, LINEs, and SINEs). Of the 318,589 SVs, 117,066 were located in the genic (including exonic, intronic, the 2-kb upstream and downstream regions, 3′UTR, and 5′UTR) and 211,602 were in the intergenic regions. Among the 32,942 genes in the rice genome, we identified 21,176 genes that were highly conserved and had no SV in their entire genic regions (including the 2-kb upstream and downstream regions). Approximately 91% of genes (19,333) exhibited no variation in their coding sequences (CDSs). There were 1385 genes with no frame shift indels; these genes, in combination with the genes without amino acid alterations, were categorized as structurally conserved genes. However, approximately 12,085 of the 32,942 genes had large SVs in their entire genic regions (Additional file 2: Table S13, S16).

We compared the GC content and the numbers of SNPs and TEs in the ~ 800 kb flanking regions of all identified SVs, the diversity for both SNPs and TEs decreased with the distance increased, but the GC content was slightly lower in SV and its proximal upstream regions (Fig. 2f). To elucidate how the SVs affect their surrounding genes, we examined the gene expression patterns through the RNA-seq data collected from the leaf, spikelet, flower, and grouting tissues of 13 wild and cultivated rice accessions and identified differentially expressed genes (DEGs). In total, 9859 genes were expressed in all tissues, and 41,818 genes showed specific expression in leaf (5883), spikelet (6938), flower (6058), or filling (6673) tissues. We found that there were much more DEGs in the downstream regions of SVs than in the upstream regions (Fig. 2g).

### Contribution of SVs during rice domestication

To provide a comprehensive SV resource for cultivated and wild rice, we then genotyped a rice genome diversity panel consisting of 169 wild and 480 cultivated rice accessions based on Illumina WGS data (Additional file 2: Table S14). Of all the successfully genotyped accessions, we discovered 310,833 of all the SVs (328,723) in at least one rice genome except reference genome. According to the SVs identified, these rice samples were classified into three major groups, *japonica*, *indica*, and wild rice groups (Additional file 1: Fig. S8). The SV-inferred phylogenetic relationships among these accessions were mostly consistent with those obtained from previous studies based on SNPs [14, 23].

**Fig. 3:**
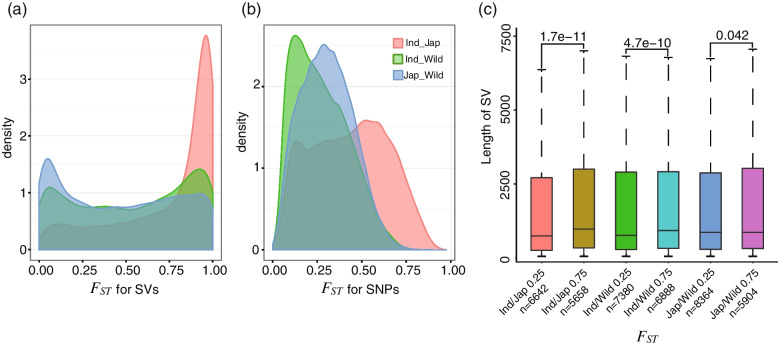
Feature of SVs associated with domestication. **a** Distribution of *F*_*ST*_ values between *O. rufipogon*, based on SVs within 20-kb windows, with *japonica* and *indica*. The corresponding plots for SNPs are provided in **b**. **c** Box plots of SV length under the selective sweeps detected by *F*_*ST*_ analyses or not

Theoretically, genes contributing to domestication can be detected as regions to mark chromosome divergence between cultivated and wild rice. Here, *O. rufipogon* was compared to both *japonica* and *indica* through genome-wide estimation of SV and SNP divergence in fixed 20-kb windows. Across the genome, mean *F*_*ST*_ estimates were considerably higher for SVs (*japonica*–*rufipogon*: 0.448 ± 0.112; *indica*–*rufipogon*: 0.495 ± 0.111) than for SNPs (*japonica*–*rufipogon*: 0.307 ± 0.018; *indica*–*rufipogon*: 0.271 ± 0.02). The distribution of *F*_*ST*_ values calculated by SVs and those calculated by SNPs was significantly different. There were two peaks in the distribution of *F*_*ST*_ values calculated by SVs (Fig. 3a, b). Approximately 24% of the *F*_*ST*_ values for SVs identified between *japonica* and *rufipogon* were > 0.75, and 34% were < 0.25. Of the *F*_*ST*_ values for SVs identified between *indica* and *rufipogon*, 28% were > 0.75 and 30% were < 0.25. The average length of SVs in the regions with *F*_*ST*_ > 0.75 was larger than that in the regions with *F*_*ST*_ < 0.25 (Fig. 3c). These results indicated that SVs are typically fixed polymorphism between wild and cultivated rice populations.

**Fig. 4:**
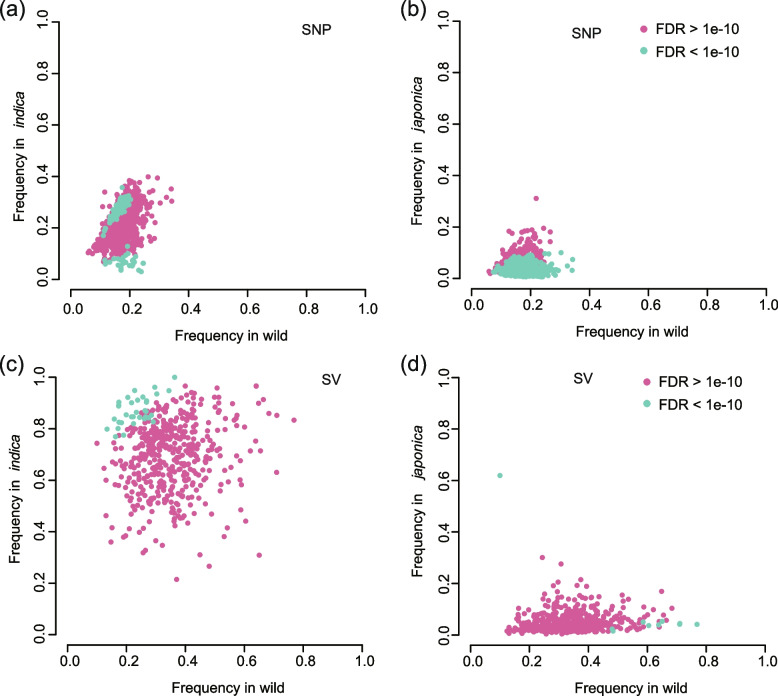
Scatter plots showing SNP (**a**,**b**) and SV (**c**,**d**) occurrence frequencies in *japonica* and *indica* subspecies

Subsequently, the two cultivated rice subspecies were compared to *O. rufipogon* and the top 5% *F*_*ST*_ windows (23 of 1230 blocks across the *japonica* genome and 26 of 1233 blocks across the *indica* genome) for both SVs and SNPs were ranked. Only 1.87% of *F*_*ST*_ blocks fell in the top 5% for both SVs and SNPs, and only 3.72% of the top 5% XP-CLR score windows (68 of 1827 windows calculated by SNPs and 62 of 1572 windows calculated by SVs) fell in both SVs and SNPs (Additional file 1: Fig. S11). The differences in SNP and SV allele frequencies between the two cultivated rice subspecies and *O. rufipogon* were further compared. In *indica*, a total of 105 domestication sweeps covering 8.3% (31.6 Mb) of the genome were detected with SNPs, while 145 domestication sweeps covering 12.6% (48.2 Mb) of the genome were detected using SVs (Fig. 4). There was a 12.4-Mb overlap between domestication sweeps identified using SNPs and those using SVs. In *japonica*, a total of 178 domestication sweeps covering 14.3% (54.1 Mb) of the genome were detected with SNPs (Fig. 4a, b), while 145 domestication sweeps covering 7.2% (27.6 Mb) of the genome were detected using SVs (Fig. 4c, d). There was only a 11.7-Mb overlap between domestication sweeps identified using SNPs and those using SVs. These results showed that the evolutionary history of SNPs and SVs between cultivated and wild rice populations is different.

### Genome-wide association study between SVs and traits

Sequence resolution of such major SV alleles would provide a critical, initial step for the development of a canonical rice reference genome. To further functionally characterize these SVs in the rice genome, GWASs were employed for nine agronomically important traits (Additional file 1: Fig. S9), including grain length (GL), grain width (GW), flag leaf length (FLL), flag leaf width (FLW), primary branch (PB), plant height (PH), secondary branch (SB), and 1000 grain weight (TGW). To characterize the associated SVs, a set of 75,606 large SVs (> 50 bp) with MAF > 0.01 was used for GWASs performed separately. This yielded reliable candidate SVs, which were employed for identifying genes regarding the nine traits (Additional file 1: Fig. S10; Additional file 2: Table S15). As shown in Manhattan plots (Additional file 1: Fig. S10), 413 significantly associated SVs (defined by –log_10_p > 3) were identified, involving 361 genes (Additional file 2: Table S15).

**Fig. 5:**
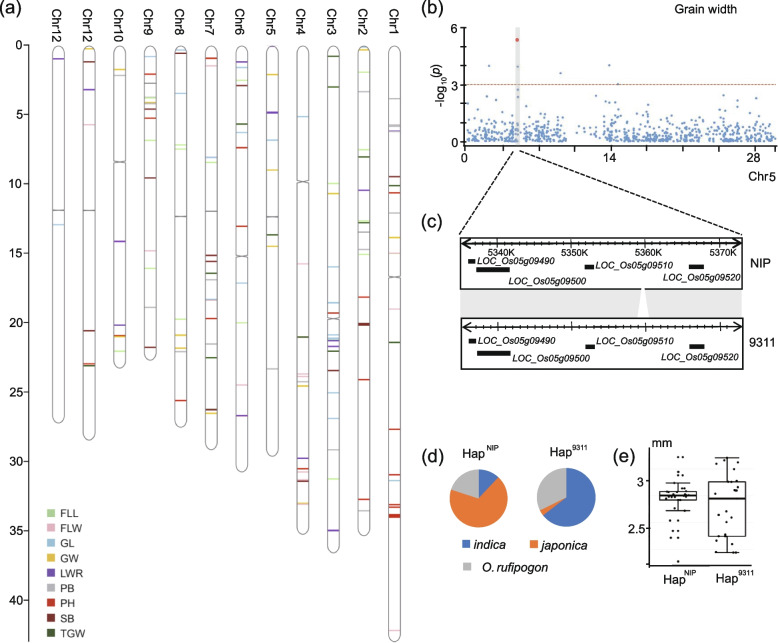
GWAS of grain length, grain width, the ratio of grain length to grain width, grain weight, primary branch, secondary branch, plant height, flag leaf length, flag leaf width, and the angle of flag leaf for 649 rice accessions based on SVs. **a** Plots of significant associated sites with the nine traits on chromosomes. **b** Manhattan plot of grain width using SV-GWAS on chromosome 5. **c** SV results in the presence and absence of Nipponbare (NIP) and 9311. **d** Haplotype frequencies in cultivated and wild rice. **e** Comparison of the grain width variation between the two haplotypes of the 1200-bp SV

Rice grain size plays a crucial role in the determination of grain quality and yield. We identified 83 quantitative trait loci (QTLs) involving four grain size-related traits (20 for GL, 16 for GW, 22 for LWR, and 24 for TGW; Fig. 5a; Additional file 2: Table S15), which explained 8.37–53.32% of the total phenotypic variance. Among these QTLs, three were colocalized with the *GW5*/*qSW5*, *OsSWN3*, and *DTE1* genes, of which *GW5*/*qSW5* demonstrated the strongest effect on GW. Encouragingly, SV-GWAS directly detected a 1.2-kb insertion in the promoter region of *LOC_Os05g09520* (*GW5*), which was determined a causal SV between Niponbare and R498 (Fig. 5b). Among the 649 rice genomes, *indica* accessions (94%) had insertions upstream of the *LOC_Os05g09520* promoter (Fig. 5c). Accordingly, these accessions with Hap^NIP^ exhibited a significantly larger GW than others with Hap^9311^ (Fig. 5d, e). These results indicated that SV-GWAS is complementary to SNP-GWAS in detecting the association with SV-caused phenotypes.

**Fig. 6:**
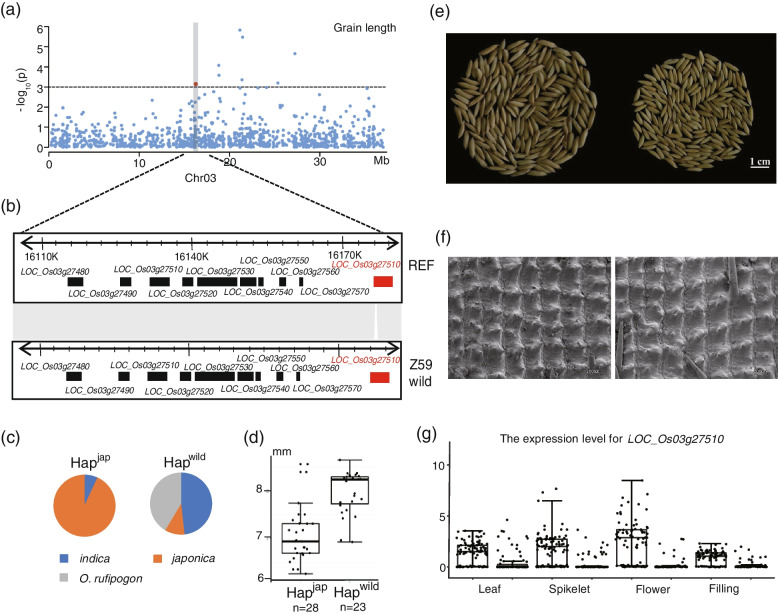
GWAS on grain length in natural and BCF_5_ rice populations. **a** Manhattan plot of grain length using SV-GWAS on chromosome 5. **b** SV results in the presence-and-absence of Nipponbare (NIP) and wild rice (SL1). **c** Haplotype frequencies in cultivated and wild rice. **d** Comparison of the grain length variation between the two haplotypes of the 8598-bp SV. **e** Phenotypic characterization of two lines in the BCF_5_ population. **f** Scanning electron microscopic analysis of the outer spikelet hull surfaces of two lines in the BCF_5_ population. **g** Expression profiling of *LOC_Os03g27510*

SV-GWAS detected eight consistent peaks associated with GL in chromosome 3. However, the peak presence and absence variations (PAVs) were located within one annotated coding gene, *LOC_Os03g27510*, which encodes a serine carboxypeptidase family protein (Fig. 6a). The peak SV (824 bp; SV824) was in the tenth exon of *LOC_Os03g27510* (Fig. 6b). We then analyzed the haplotypes of this SV, and the Hap^jap^ was identified in 93% *japonica* accessions and 6% *indica* accessions but was not present in wild rice accessions (Fig. 6c). GL was shorter in cultivated rice accessions with Hap^jap^ than those with Hap^wild^ (Fig. 6d). To unravel the effect of these SVs on GL, we constructed a BC_1_F_5_ population segregating for SV824 but was fixed. Y283 and Y195 were two lines with significant difference in grain length. Longer grain length co-segregated with Hap^wild^. Y283 with Hap^jap^ was slightly shorter with Y195 with Hap^wild^ (Fig. 6e). Cytological observation was carried for spikelet hulls to examine Y283 and Y195 in detail. Scanning electron microscopy observation illustrated more epidermal cells in the spikelet hull surface in Y283 with Hap^jap^ compared to Y195 with Hap^wild^ (Fig. 6f). Moreover, the expression analysis based on transcriptome also demonstrated that all accessions with Hap^wild^ were expressed approximately 5- or 10-fold higher than in accessions carrying Hap^jap^ (Fig. 6g). These observations suggested that this SV affects grain size.

## Discussion

The roles played by SVs in plant domestication and the formation of phenotypic diversity have not yet been widely explored in large-scale populations [3]. In this study, we compared 13 representative species of long-read sequencing data for cultivated and wild rice varieties and obtained relatively accurate and complete SV datasets for cultivated and wild rice accessions, especially in TE-rich regions. These variations were identified not only based on long-read resequencing and short-read resequencing. Many intraspecific variations were found; specifically, in the application of four pieces of long-read sequencing data of wild rice, we found 157,369 SVs were unique to wild rice and may have been lost during rice domestication. We revealed that more than 12,085 genes in the rice genome were related to large-effect mutations, and SVs also had significant influences on the expression of their surrounding genes.

We used 649 for cultivated and wild rice accessions to classify the 318,589 identified SVs and found that the overlap between selective sweeps of SVs and SNPs in the rice genome was less than 2%. The similar result was also observed in a comparative analysis on peach domestication [24]. Moreover, we further revealed that the selected distribution of SVs was bimodal and that of SNP was unimodal. We speculated that about 30% of SVs had a low frequency of random occurrence in the rice genome, but there were more than 25% of SVs that may contribute greatly to the major differentiation between the cultivated and wild rice populations. A large SV region in the genome indicates a low recombination, which will accelerate the fixation of favorable variations [7].

GWAS leverages the fact that SNPs can be mapped to a relatively accurate genomic interval [25, 26]. However, research regarding GWAS based on SVs to date is relatively less explored than other variants in plants [27, 28]. Considerable evidence has been established from rice molecular and genetic studies to demonstrate that SVs are able to result in major phenotypic variances [29, 30], which suggests that SVs may constitute a valuable source of variations for GWASs in rice. Consistent with this prediction, we found 413 SVs that were related to the formation of the diversity of agronomically important traits in cultivated and wild rice populations. Studying SVs in plant populations has become the frontier of plant genomics; therefore, we employed the SV-GWAS as a new approach to screen candidate genes related to key agronomic traits. Compared with the traditional SNP-GWAS, although the SV-GWAS yields fewer significant associations, it can focus on the missing sites in the reference genome and acquire more accurate information [27, 28, 31]. For example, we found an SV on chromosome 3, which may disrupt the formation of a serine carboxypeptidase family protein. A previous study identified a gene (*GS5*) on chromosome 5 of the rice genome and pointed out that natural variation in *GS5* plays a critical role in the regulation of rice yield and grain size. We found a new gene encoding a serine carboxypeptidase family protein may be related to this newly discovered SV. In future research, it would be useful to directly apply SV markers to the GWAS to test the genomic predictive value in rice breeding programs. Although our study captures representative genomes of cultivated and wild rice, it cannot capture wider genetic diversity of the species [3]. Because only high-confidence SV calls were retained, our results may be prone to false negatives. Nevertheless, we have taken a very important step in elucidating the effect of SVs on rice domestication and phenotypic diversity. The identified SVs provide a new resource for future research on the domestication history of cultivated rice and the formation of agronomic trait diversity. In addition, we have developed an open, user-friendly genome information platform (www.ricegermplasmgenome.com) for the users to easily access these data. This will largely accelerate the utilization of wild resources with SVs, such as research on gene mapping, GWAS, gene cloning, and marker-assisted selection.

## Conclusions

This study provided an integrated map of genome SVs by 12 whole rice genomes and resequencing 648 rice accessions. We identified and analyzed the variants of SVs on the genome and the association with agriculture traits during rice domestication. A GWAS approach using SVs found more efficient traits than SNPs in identifying candidate causal variants. This suggested candidate genes responsible for the rice grain-related traits, such as grain weight and grain length. The function of *LOC_Os03g27510* was observed to associate with rice grain length, while the candidate gene for grain width was confirmed to be *GW5/qSW5*, as previously reported. The integrated SV map provides a valuable resource for future genomic research in rice. In addition, the significant association signals identified for the 9 agronomic traits provide valuable candidates for the genetic improvement of rice.

## Materials and methods

### Plant materials

To cover a wide range of rice genetic diversity, a total of 1504 rice accessions (1071 cultivated rice and 433 wild rice accessions), which were sampled globally and represent a full range of geographical distribution (Additional file 1: Fig. S1a), were used. Each rice accession was sequenced on an Illumina platform with a coverage depth of > 7×. After mapping the clean reads to the *O. sativa* ssp. *japonica* Nipponbare (hereafter Nipponbare) genome [32], we identified a total of 12,839,501 SNPs. Phylogenetic analyses using these SNPs classified the 1504 rice accessions into seven major groups including two wild rice groups and five cultivated rice groups (Additional file 1: Fig. S1b). We selected 80 accessions for genome survey (Additional file 2: Table S1) on the basis of the above phylogenetic tree. Based on the phylogenetic tree and genome survey results (Additional file 2: Table S2), we also selected 13 rice accessions (including four wild rice and nine rice cultivars) in addition to Nipponbare to establish representative samples for de novo genome assembly. Moreover, the 649 most represented accessions (480 cultivated and 169 wild rice accessions), in terms of phylogenetic relationship and geographic distribution (Additional file 2: Table S3), were selected to genotype SVs.

### Genome survey

DNA was isolated from leaf tissues using a Plant Genomic DNA kit (Tiangen, Beijing, China). DNA contamination and degradation were checked by electrophoresis (1% agarose gel). DNA purity and concentration were measured by a NanoPhotometer spectrophotometer (IMPLEN, CA, USA) and a Qubit 2.0 fluorometer (Life Technologies, CA, USA), respectively. Approximately 1.5 μg DNA per sample was employed for the construction of Illumina paired-end (150 bp with an insert size of 400 bp) sequencing libraries. The library was constructed with a TruSeq Nano DNA HT Sample Preparation Kit (Illumina USA) according to the manufacturer’s protocols and sequenced on an Illumina HiSeq X10 platform.

Raw reads were quality controlled (QC) with in-house C scripts. QC procedures included removing (1) reads with ≥ 10% ambiguous bases (N); (2) reads having > 50% bases with a Phred quality score < 5; (3) reads having > 10 nt aligned to the adapter, allowing ≤ 10% mismatches; and (4) putative PCR duplicates. *K*-mer analysis (*k* = 17) was performed using the clean reads with Jellyfish v.2.2.6 (parameters: jellyfish count -m 17 -s -t -o -C) [33]. The ratio of total number of k-mers to its expected depth [34] was used to estimate genome size. Heterozygosity was inferred from the position of major and minor peaks in the k-mer depth distribution curve [35]. Pre-assembly on these reads was performed with Nextdenovo [36], and GC content was calculated in a sliding window of 2 kb along the assembled contigs.

### Oxford nanopore sequencing and contig assembly

Leaf tissue samples were used to isolate genomic DNA with a Genomic DNA extraction kit (Qiagen, USA) according to the manufacturer’s instructions. DNA concentration and purity were determined by a Qubit 3.0 fluorometer (Invitrogen, USA) and a Nanodrop One UV-Vis spectrophotometer (Thermo Fisher Scientific, USA), respectively. DNA fragments > 30 kb were selected using BluePippin electrophoresis (Sage Science, USA), and their ends were repaired, A-tailed, and ligated with sequencing adapters provided in the SQK-LSK109 kit (Oxford Nanopore Technologies). The established DNA library was quantitatively detected by a Qubit 3.0 fluorometer (Invitrogen, USA) and sequenced on a PromethION sequencer (Oxford Nanopore Technologies, UK).

The raw subreads obtained from Oxford Nanopore sequencing were filtered by fastp v0.12.6 with default parameters [37]. The filtered data were firstly error-corrected by Nextdenovo (read_cuoff = 3k, blocksize = 2g, seed_cutoff = 25k) and then assembled by Smartdenovo (wtpre -J 3000, wtzmo -k 21 -z 10 -Z 16 -U -1 -m 0.1 -A 1000, wtclp -d 3 -k 300 -m 0.1 -FT, wtlay -w 300 -s 200 -m 0.1 -r 0.95 -c 1) (https://github.com/Nextomics/NextDenovo). The Illumina short reads generated for genome survey were mapped to the assembly with BWA [38] to further improve the base accuracy of the assembly, which was then polished three times with Pilon [39].

### Hi-C sequencing and pseudo-chromosome construction

The Hi-C library was constructed following the procedure described previously [40] with slight modifications for improvement. Briefly, fresh leaf samples were cut into 2-cm pieces and fixed in nuclei isolation buffer containing 2% formaldehyde. The fixed tissue was frozen in liquid nitrogen and ground to fine powder. Afterward, nuclei isolation buffer was added to produce nucleus suspension. The purified nuclei were digested with *Hind*III (100 units) and marked by incubating with biotin-14-dCTP. The ligated DNA was sheared into 300−600 bp fragments, blunt-end repaired, and A-tailed. Then, purification was carried out through biotin-streptavidin-mediated pull-down. Finally, the Hi-C library was quantified and sequenced on an Illumina HiSeq platform (Illumina, San Diego, CA, USA) to obtain 150-bp, paired-end reads.

Hi-C raw data QC was conducted with Hi-C-Pro v2.8.0 [41]. Sequences with a quality score < 20, adaptor sequences, and sequences < 30 bp were removed by fastp v0.12.6 [37]. The clean reads were then mapped to the draft genome assembly with Bowtie2 v2.3.2 to obtain the uniquely mapped reads [42]. Combined with the valid interaction pairs, pseudo-chromosome assembly was subsequently constructed using the LACHESIS pipeline [43].

### Iso-seq on PacBio sequel and RNA-seq on Illumina NovaSeq platform

Total RNA was isolated from five rice tissues (i.e., flowers, spikes, stems, leaves, and roots) by the TRNzol Universal kit (Tiangen) following the manufacturer’s standard procedure. RNA concentration and purity were detected with a Qubit 3.0 fluorometer (Invitrogen, USA) and a NanoDrop One UV-Vis spectrophotometer (Thermo Fisher Scientific, USA), respectively. The values of RNA integrity number (RIN) and the ratio of 28S/18S for the extracted RNA were detected using an Agilent 2100 bioanalyzer (Agilent technologies, USA).

For Iso-seq, about 2.0 μg of equally mixed RNA from the five tissues was utilized for RNA library preparation. The library was generated using the SMRTbell Template Prep Kit 1.0 as described previously [44] and sequenced on a PacBio Sequel platform (Pacific Biosciences). For RNA-seq, the five tissues were separately used for RNA sample preparation. The library was generated with a TruSeq RNA Library Preparation Kit (Illumina, USA) following the manufacturer’s protocols. The index was added to attribute reads to each sample, and the clustering of the indexed samples was performed on a cBot Cluster Generation System by TruSeq PE Cluster Kit v3-cBot-HS (Illumina, San Diego, CA, USA) following the manufacturer’s procedures. After clustering, the libraries were sequenced on an Illumina NovaSeq platform, and 150-bp, paired-end reads were obtained.

### Genome annotation

To predict repeat sequences, we employed de novo prediction with RepeatModeler v5.8.8 and homology-based searches against the Repbase database by RepeatMasker v3.2.1 (http://www.repeatmasker.org). To predict gene models, de novo prediction, homology-based search, and transcriptome-based analysis were adopted. For de novo gene prediction, we used the Augustus [45] and Snap [46] pipelines. For homology evidence, we searched homologous genes against the proteins of *Arabidopsis thaliana*, *Zea mays*, *Hordeum vulgare*, *O. sativa*, and *Physcomitrella patens* with GeneWise [47], separately. For transcript evidence, we obtained full-length transcripts from Iso-seq and Trinity-assembled transcripts from the RNA-seq data of the five rice tissues using the annotation pipeline PASA v2.0.2. We obtained the final gene set by integrating the results from the above three methods with Evidence Modeler (https://github.com/EVidenceModeler) and removing genes containing transposon elements with TransposonPSI (http://transposonpsi.sourceforge.net/).

### Structural variation identification

The smartie-sv pipeline (https://github.com/zeeev/smartie-sv) was used to call SVs (deletions and insertions; > 50 bp). We then adopted two rounds of filtering to the initial SV set. First, the SVs that were < 50 bp or within the centromeric region were removed. Second, regions (1-Mb window) with > 50 alignments were omitted. Third, contigs < 200 kb were removed. Furthermore, seven SVs (50 bp–2 kb) were verified by Sanger sequencing and good collinearity in the flanking sequences of SVs was shown between two genomes. The 386,014 SVs should therefore be acceptable as an initial dataset, in spite of some false positives. To obtain more reliable SV sets, All 12 assemblies were aligned to the Nipponbare genome using Mummer (v 4.0) with the parameters: -l 50 -c 100. The raw alignments results were further filtered using delta-filtre with parameters: -m -i 90 -l 100. The resulting filtered delta files were used to detect structural variations using the SyRI pipeline with default parameters; 113,472 SVs have been established by this method. After combined with smartie-sv results, 80,290 SVs (70.76% of 113,472 SVs) are identical or overlapping with the 318,589 SVs established by smartie-sv pipeline. Based on these results and analyses, the smartie-sv SVs set is selected for further analysis.

We combined SVs identified across 649 rice accessions with the “bayesTyperTools combine” module in BayesTyper v1.3.1. The whole-genome sequencing (WGS) reads of each accession were trimmed based on BayesTyper v1.3.1 by default settings. The “bayesTyper genotype” module was applied to assess the genotype of each rice accession based on k-mers of Illumina short reads, and the outputs were merged by BCFtools v1.8 (--force-samples --filter-logic x --info-rules ACP: max). After genotyping SVs in each genome compared with Nipponbare, the individual results were merged into a single hapmap file, in which 324,750 SVs were genotyped in at least one rice accession. Finally, unique polymorphic SVs with MAF > 0.05 were obtained.

### Statistical analysis

With 2000 bp taken from the upstream and downstream of each SV and a 200-bp bin (0–200, 201–400, 401–600, 601–800, and 801–1000 bp), the number of SNPs, TEs, and genes, as well as the GC content in each bin, were determined. Each chromosome was separately counted, and the data of 12 chromosomes were obtained. The frequency of Alt allele at each SNP locus in the wild rice and cultivated rice (*indica* and *japonica*) was calculated, and the average frequency of each window (window size was 10 kb) was determined. With 30 windows taken as a group, the difference in frequency distribution between the two groups was compared, and *t*-test was utilized to estimate significant *p* values.

### Genome-wide association studies

A genome-wide association study (GWAS) based on SVs was employed for flowering time, seed weight, and silique length with the mixed linear model (MLM) in GEMMA v0.98 software. The effective number (*n*) of independent SNPs and SVs were calculated the PLINK software (www.cog-genomics.org/plink2; v1.9). Based on these methods, 317,102 high-confidence independent SNPs and 2378 high-confidence independent SVs were identified. We defined the whole-genome significance cutoff as the Bonferroni test threshold, which was set as 1/n to control the genome-wide type I error rate.

## Supplementary Information

**Additional file 1: Fig. S1.** (a) Geographic origins of cultivated and wild rice accessions in this study. (b) SNP-based phylogenetic tree based on short-read sequencing of more than 600 cultivated and wild rice accessions. Major taxonomic groups are marked by colored lines along the circumference. Colored dots indicate a subset of the 80 accessions selected for genome survey. **Fig. S2.** Chromosome karyotyping of JX1 (a) and SL1 (b) in meiosis period in the root tip. **Fig. S3.** Distribution of short reads mapped on corresponding genome assemblies. (a) is JX1 and (b) is SL1. **Fig. S4.** Dot plots of assembly comparisons between JX1 and Nipponbare (a) and between SL1 and Nipponbare (b). **Fig. S5.** Relationship between SVs and their surrounding genes. **Fig. S6.** Venn-like diagram presenting the numbers of SVs among *indica*, *japonica*, and wild rice. **Fig. S7.** For each SV, the distance to the telomeric (a) and centromeric regions (b) of each chromosome was calculated and divided into 100-kb bins. (c) SV densities of short/long chromosome arms in continuous 200-kb windows. Significance was tested by Fisher’s exact test; NA indicates p > 0.05. **Fig. S8.** PCA plots of cultivated and wild rice accessions based on SV diversities. The three subgroups are indicated in different colors at the top of the Fig. **Fig. S9.** Phenotypes of the rice population used for GWAS, include FLL (flag leaf length), FLW (flag leaf width), GL (grain length), GW (grain width), PB (primary branch), PH (plant height), SB (secondary branch), and TGW (1000 grain weight). **Fig. S10.** GWASs on FLL, FLW, GL, GW, PB, PH, SB, and TGW using the compressed MLM. Manhattan plots for these phenotypes. The log10-transformed P values from a genome-wide scan were plotted against the position on each of the 12 chromosomes. Quantile-quantile plot for these phenotypes. The horizontal axis shows log10-transformed expected P values, and the vertical axis indicates log10-transformed observed P values. **Fig. S11.** (a) XP-CLR scores calculated by SNPs comparing wild rice with cultivated rice. (b) XP-CLR scores calculated by SVs comparing wild rice with cultivated rice. Chromosomes are painted with either blue or orange and the black line is defined using the top 5% XP-CLR scores.

**Additional file 2: Table S1.** The results of 80 cultivated and wild rice genomes survey. **Table S2.** Summary of Nanopore Technologies sequencing data of JX1 and SL1 genome. **Table S3.** Summary of sequencing assembly data of JX1 and SL1 genome. **Table S4.** Details of the 12 pseudo-chromosomes of JX1 and SL1. **Table S5.** Summary of sequencing evaluation data of JX1 and SL1genome. **Table S6.** Repetitive elements in JX1 and SL1 genomes. **Table S7.** The information of full length transcriptome sequencing data of JX1 and SL1. **Table S8.** The transcriptome sequencing data from different tissues of JX1 and SL1 based on Illumina sequence. **Table S9.** Annotated predictions of gene structure. **Table S10.** Summary of the assembly of 13 cultivated and wild rice genomes. **Table S11.** The SV information for 13 cultivated and wild rice. **Table S12.** Sequence information for structural variation. **Table S13.** The annotation of SV. **Table S14.** The sample list for SV population analysis. **Table S15.** The significant association sites based on GWAS data. **Table S16.** 49 cloned genes overlapping with DELs (>10 kb) region.

## Data Availability

All data generated and analyzed during this study are included in this published article and its supplementary information files. Additional file 1 provides 11 supplementary figures. Additional file 2 contains 16 supplementary tables. The sequencing data, genome assemblies, and other data supporting the findings of this study have been deposited in http://124.70.162.174:8015/download/ (Biosample accessions: SAMN20799635, SAMN20799636, SAMN20799637, SAMN20799638, SAMN20799639, and SAMN20799640) and are freely available for anyone who is interested in our work.

## Abbreviations

BUSCO: Benchmarking universal single-copy orthologs
CDS: Coding sequences
DEG: Differentially expressed genes
FLL: Flag leaf length
FLW: Flag leaf width
GL: Grain length
GW: Grain width
GWAS: Genome-wide association study
LTR: Long terminal repeat
KEGG: Kyoto Encyclopedia of Genes and Genomes
MLM: The mixed linear model
NR: Non-Redundant Protein Sequence Database
PAVs: Presence and absence variations
PB: Primary branch
PH: Plant height
RIN: RNA integrity number
SB: Secondary branch
QTL: Quantitative trait loci
SNPs: Single-nucleotide polymorphisms
SV: Structural variants
TEs: Transposable elements
TGW: Grain weight
WGS: Whole-genome sequencing
