# Supplementary material for: Lost genome segments associate with trait diversity during rice domestication
Source: BMC Biol. 2023 Feb 1;21:20. doi: 10.1186/s12915-023-01512-6 (PMC9893545; doi:10.1186/s12915-023-01512-6)
Supplement: Supplementary file 1 — Additional file 1: Fig. S1. (a) Geographic origins of cultivated and wild rice accessions in this study. (b) SNP-based phylogenetic tree based on short-read sequencing of more than 600 cultivated and wild rice accessions. Major taxonomic groups are marked by colored lines along the circumference. Colored dots indicate a subset of the 80 accessions selected for genome survey. Fig. S2. Chromosome karyotyping of JX1 (a) and SL1 (b) in meiosis period in the root tip. Fig. S3. Distribution of short reads mapped on corresponding genome assemblies. (a) is JX1 and (b) is SL1. Fig. S4. Dot plots of assembly comparisons between JX1 and Nipponbare (a) and between SL1 and Nipponbare (b). Fig. S5. Relationship between SVs and their surrounding genes. Fig. S6. Venn-like diagram presenting the numbers of SVs among indica, japonica, and wild rice. Fig. S7. For each SV, the distance to the telomeric (a) and centromeric regions (b) of each chromosome was calculated and divided into 100-kb bins. (c) SV densities of short/long chromosome arms in continuous 200-kb windows. Significance was tested by Fisher’s exact test; NA indicates p > 0.05. Fig. S8. PCA plots of cultivated and wild rice accessions based on SV diversities. The three subgroups are indicated in different colors at the top of the Fig. Fig. S9. Phenotypes of the rice population used for GWAS, include FLL (flag leaf length), FLW (flag leaf width), GL (grain length), GW (grain width), PB (primary branch), PH (plant height), SB (secondary branch), and TGW (1000 grain weight). Fig. S10. GWASs on FLL, FLW, GL, GW, PB, PH, SB, and TGW using the compressed MLM. Manhattan plots for these phenotypes. The log10-transformed P values from a genome-wide scan were plotted against the position on each of the 12 chromosomes. Quantile-quantile plot for these phenotypes. The horizontal axis shows log10-transformed expected P values, and the vertical axis indicates log10-transformed observed P values. Fig. S11. (a) XP-CLR scores calculated b [file 12915_2023_1512_MOESM1_ESM.pdf]

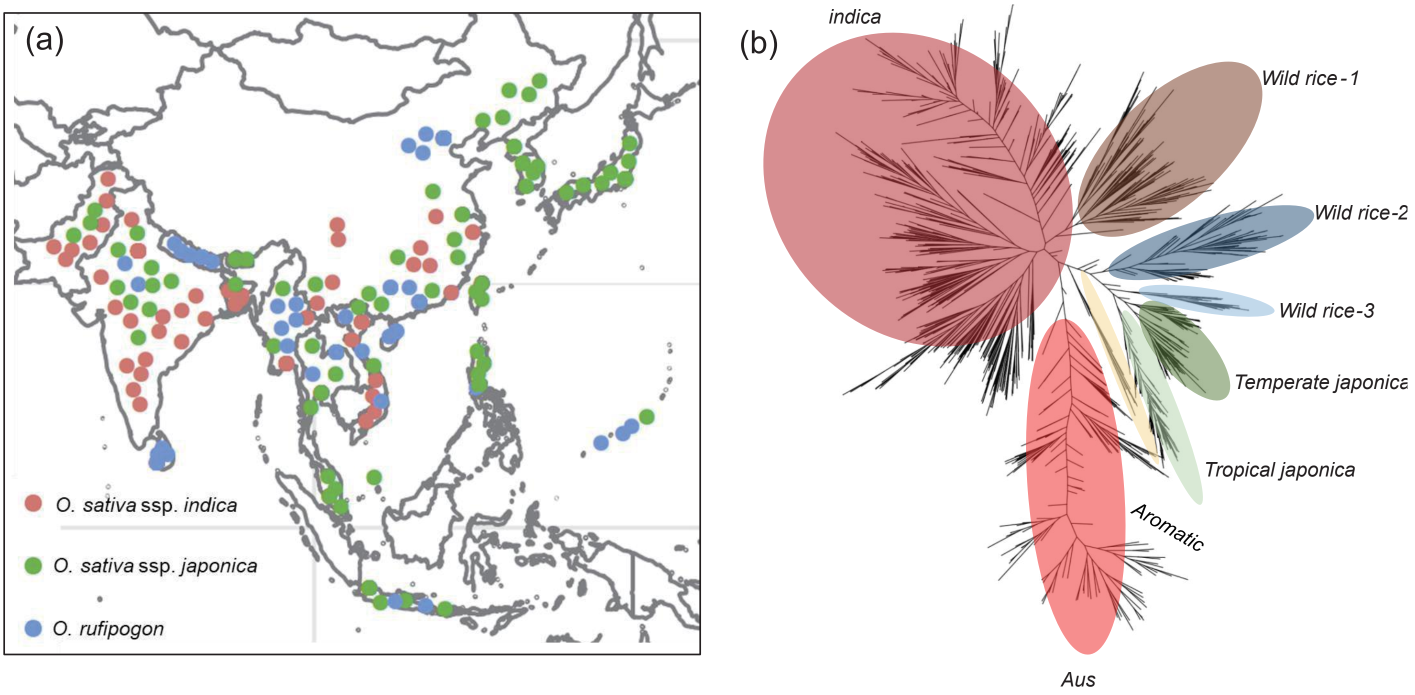

Figure S1. **(a)** Geographic origins of cultivated and wild rice accessions in this study. **(b)** SNP-based phylogenetic tree based on short-read sequencing of more than 600 cultivated and wild rice accessions. Major taxonomic groups are marked by colored lines along the circumference. Colored dots indicate a subset of the 80 accessions selected for genome survey.

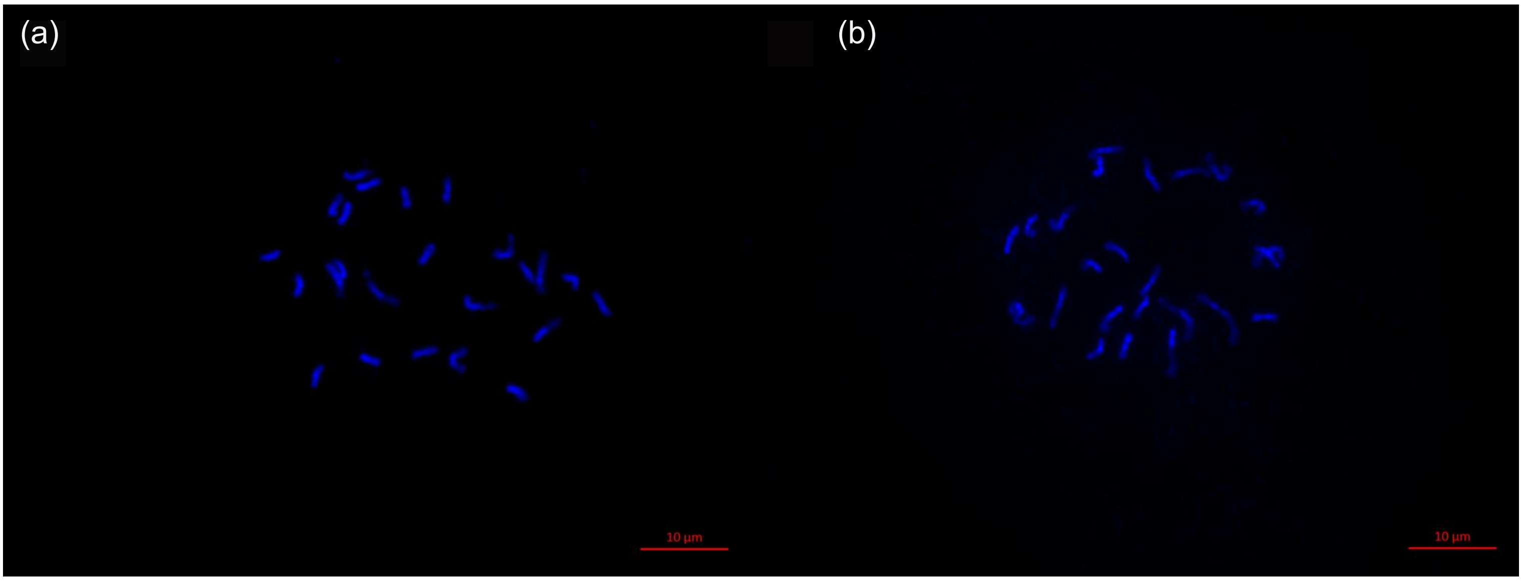

Figure S2. Chromosome karyotyping of JX1 (a) and SL1 (b) in meiosis period in the root tip.

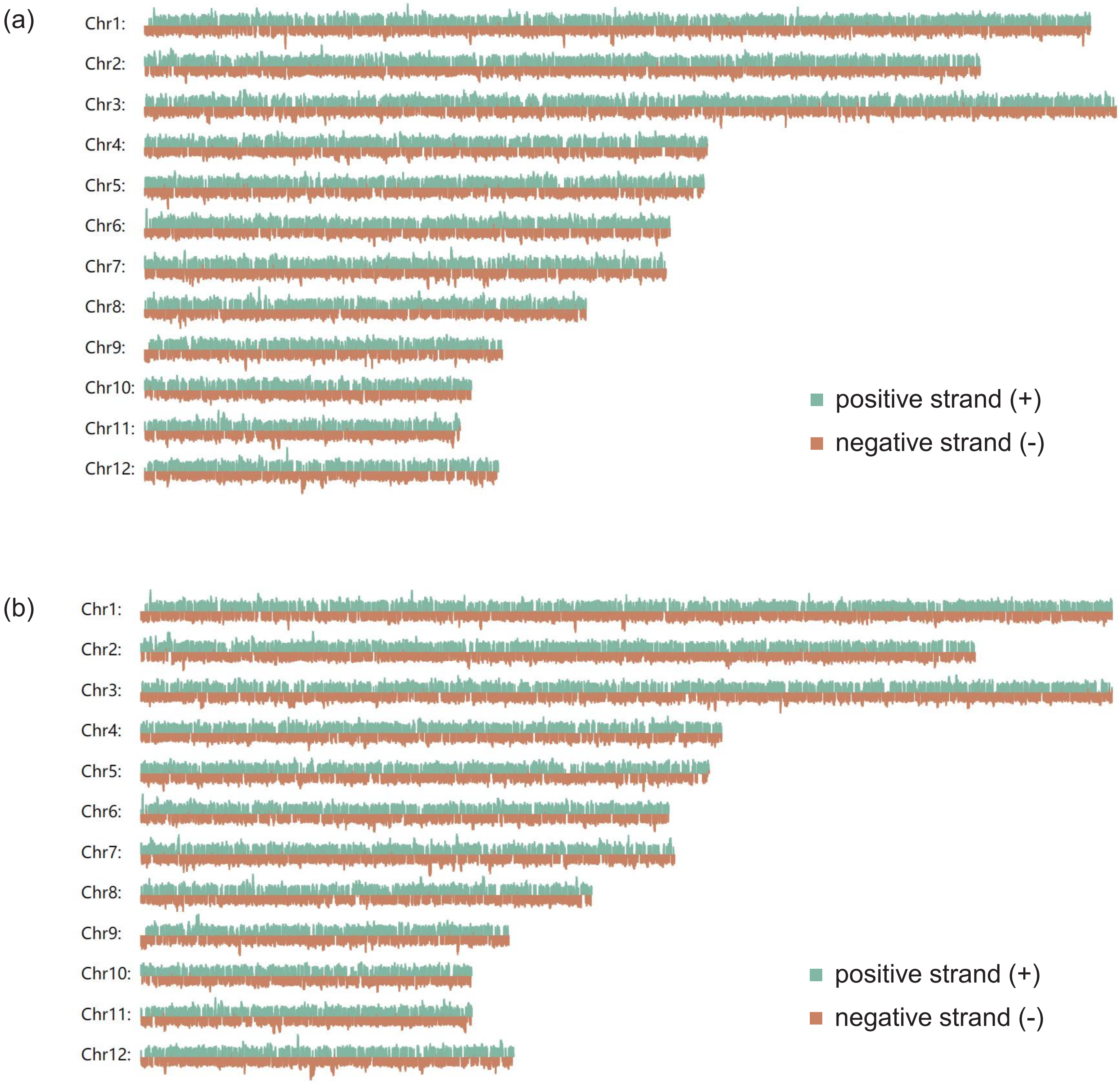

Figure S3. Distribution of short reads mapped on corresponding genome assemblies. (a) is JX1 and (b) is SL1.

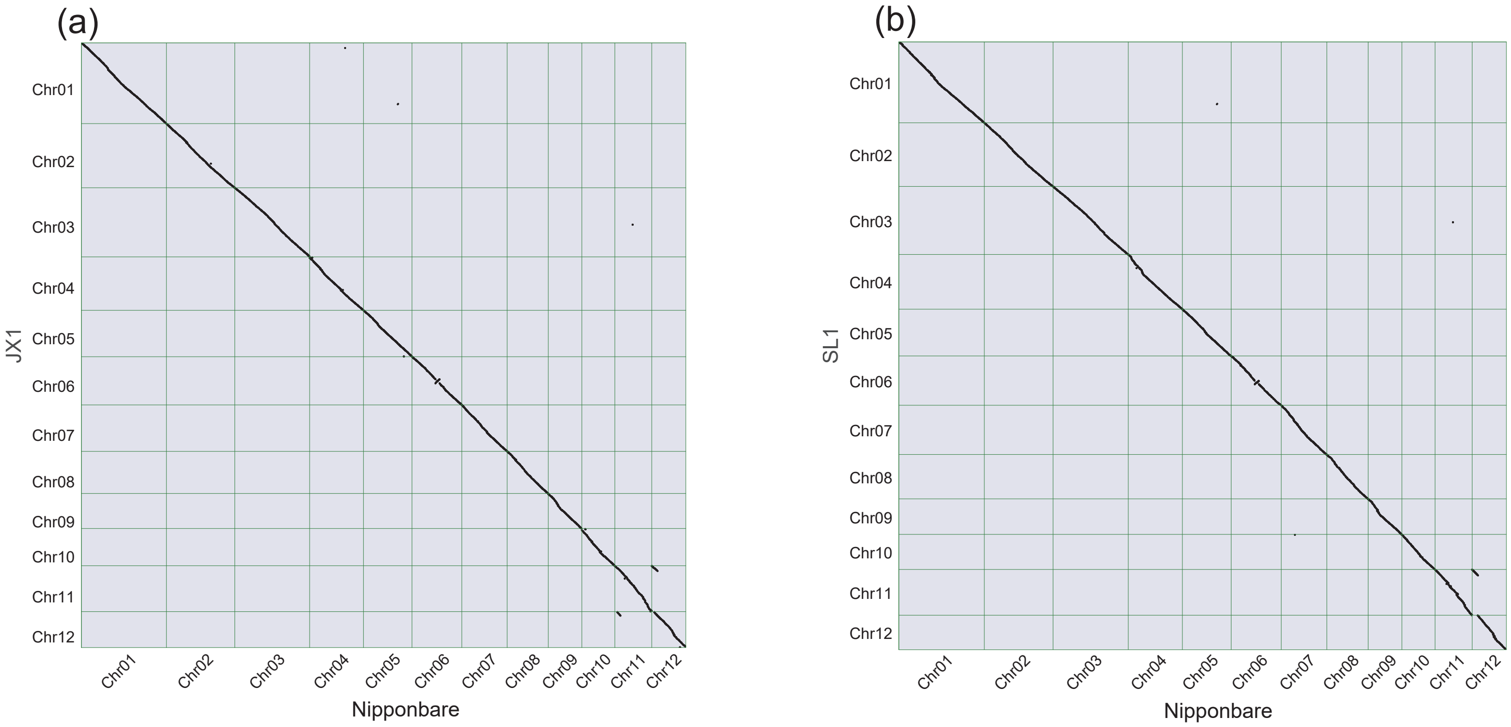

Figure S4. Dot plots of assembly comparisons between JX1 and Nipponbare (a) and between SL1 and Nipponbare (b).

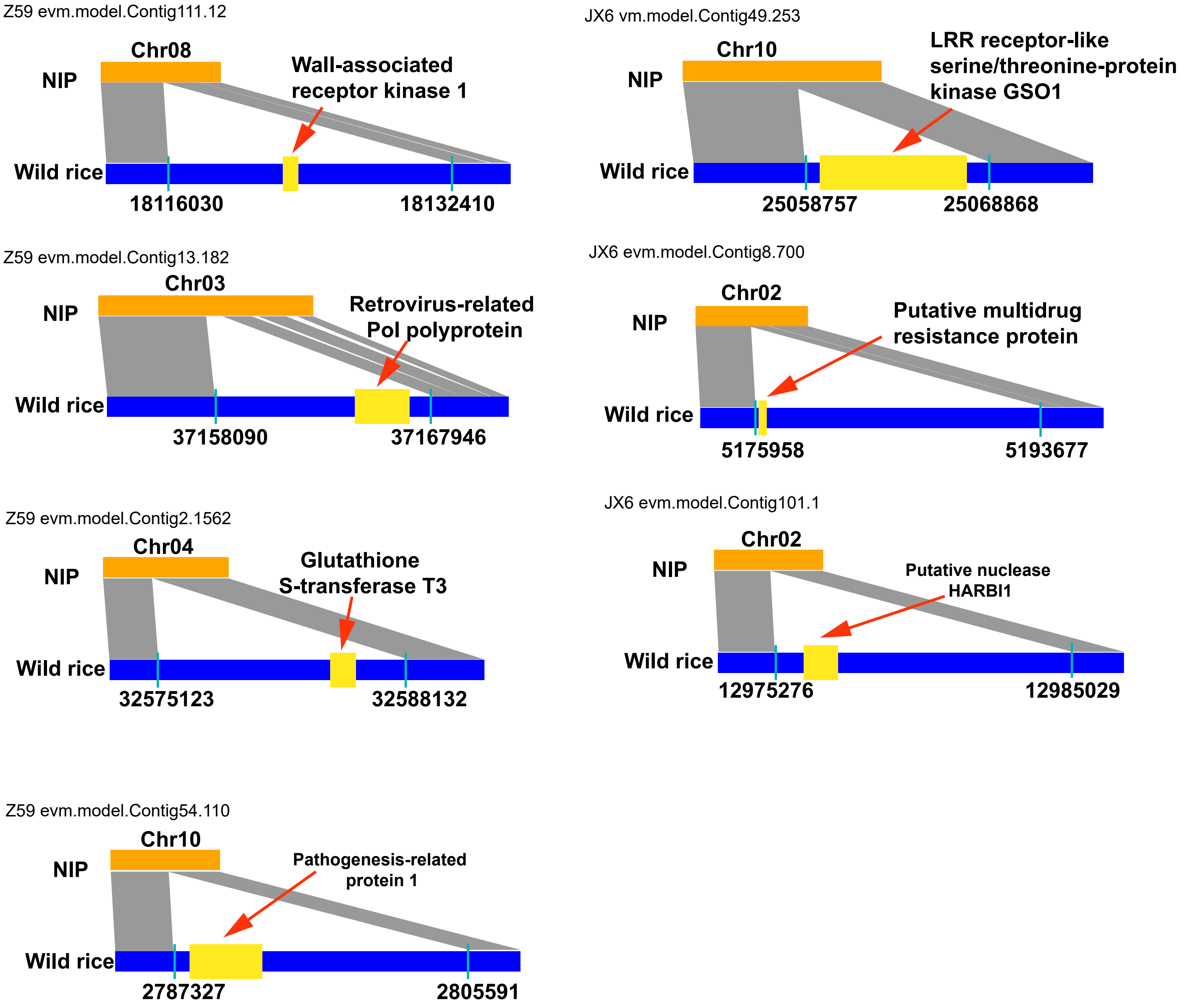

Figure S5. Relationship between SVs and their surrounding genes.

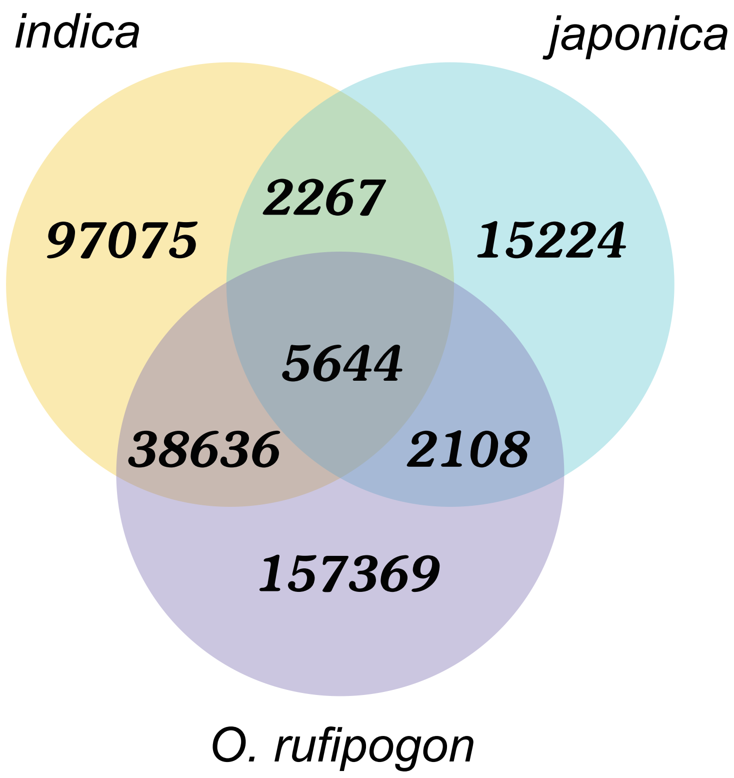

Figure S6. Venn-like diagram presenting the numbers of SVs among *indica*, *japonica*, and wild rice.

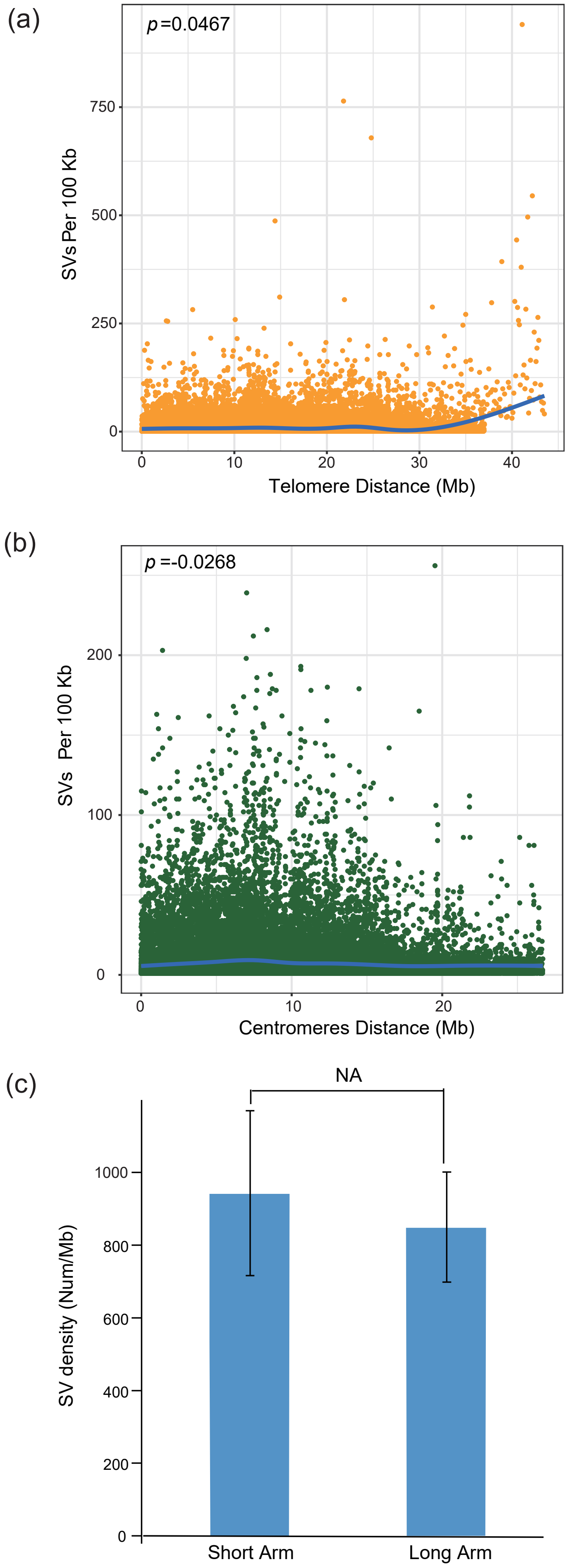

Figure S7. For each SV, the distance to the telomeric (a) and centromeric regions (b) of each chromosome was calculated and divided into 100-kb bins. (c) SV densities of short/long chromosome arms in continuous 200-kb windows. Significance was tested by Fisher's exact test; NA indicates $p > 0.05$.

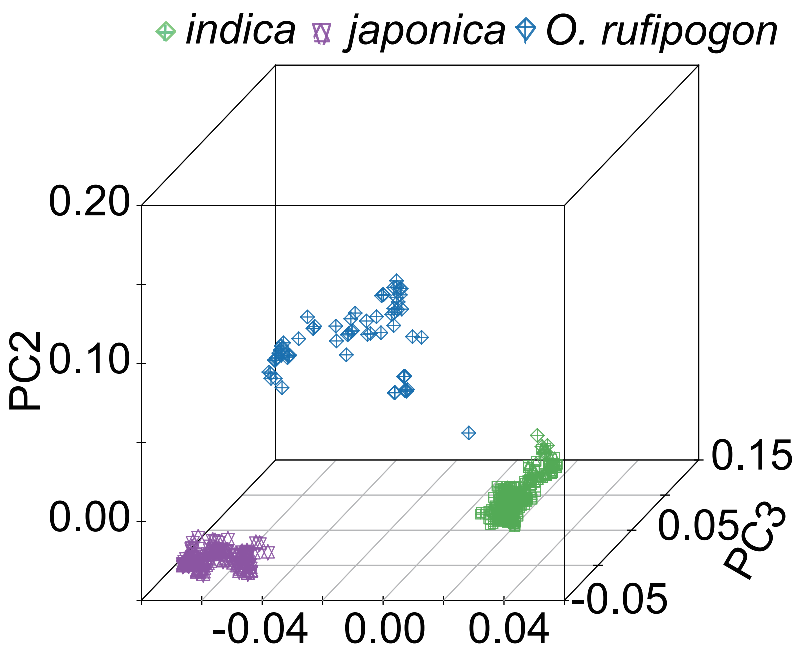

Figure S8. PCA plots of cultivated and wild rice accessions based on SV diversities. The three subgroups are indicated in different colors at the top of the figure.

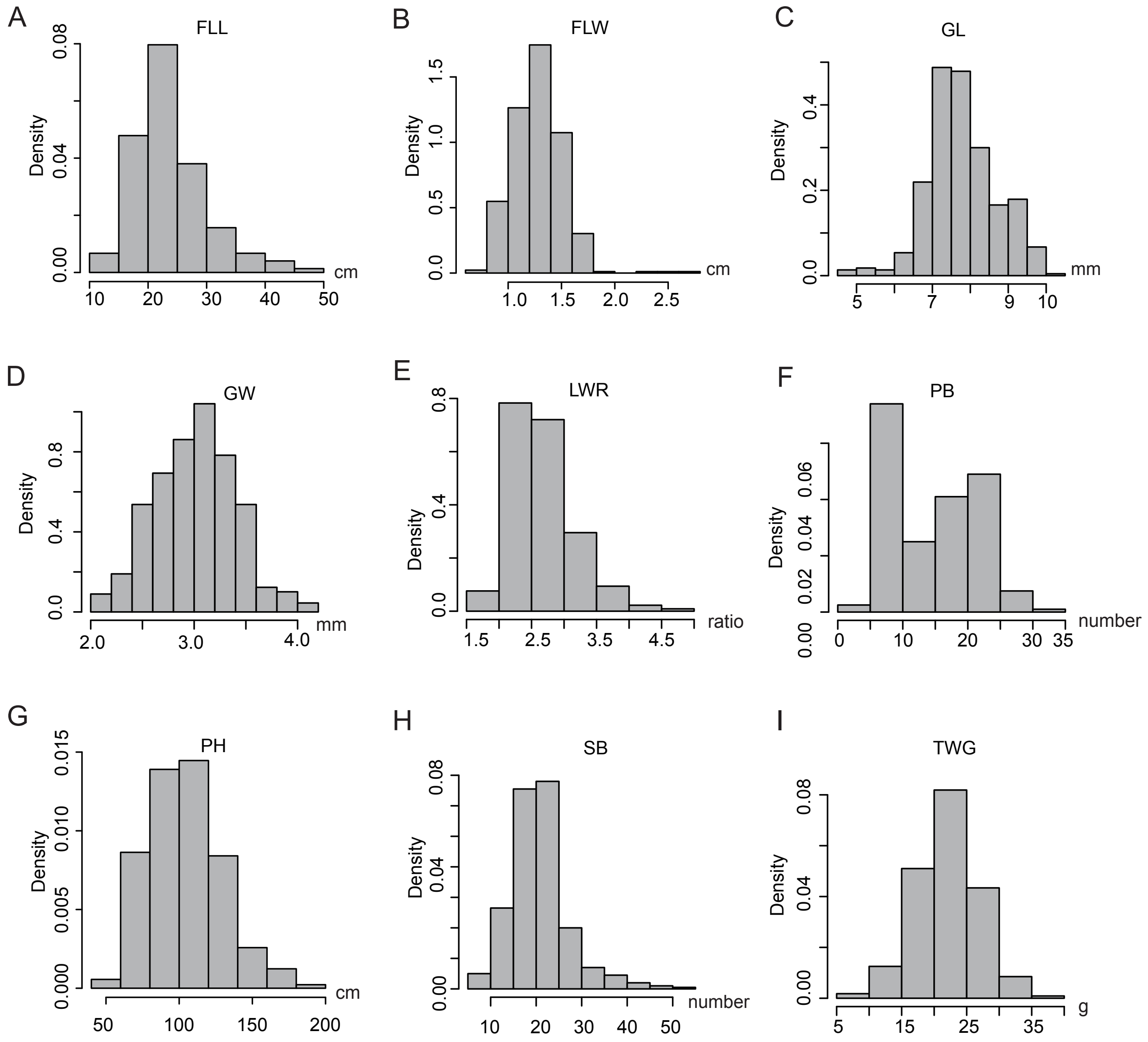

Figure S9. Phenotypes of the rice population used for GWAS, include FLL (flag leaf length), FLW (flag leaf width), GL (grain length), GW (grain width), PB (primary branch), PH (plant height), SB (secondary branch), and TGW (1000 grain weight).

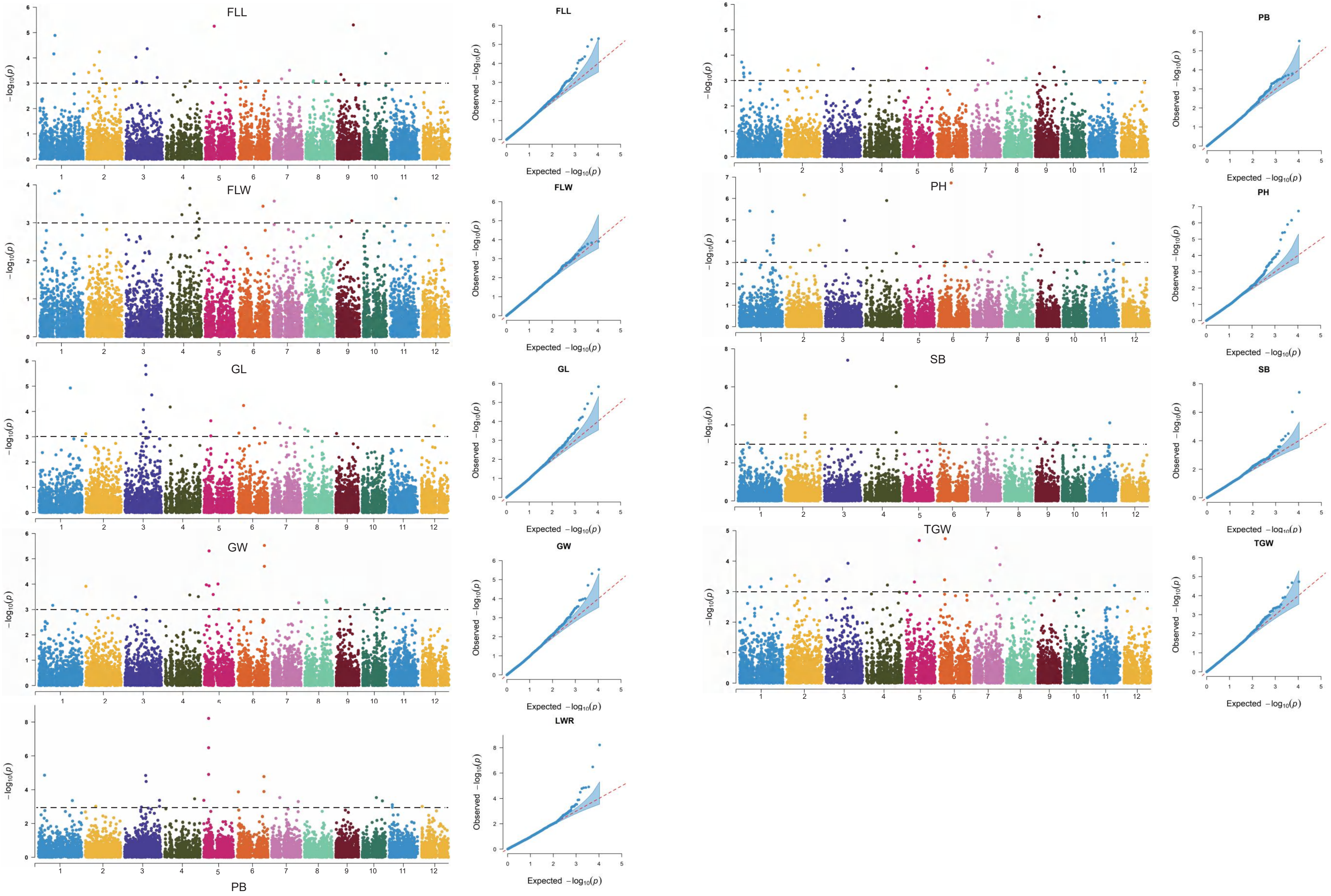

Figure. S10. GWASs on FLL, FLW, GL, GW, PB, PH, SB, and TGW using the compressed MLM. Manhattan plots for these phenotypes. The log10-transformed P values from a genome-wide scan were plotted against the position on each of the 12 chromosomes. Quantile-quantile plot for these phenotypes. The horizontal axis shows log10-transformed expected P values, and the vertical axis indicates log10-transformed observed P values.

a.

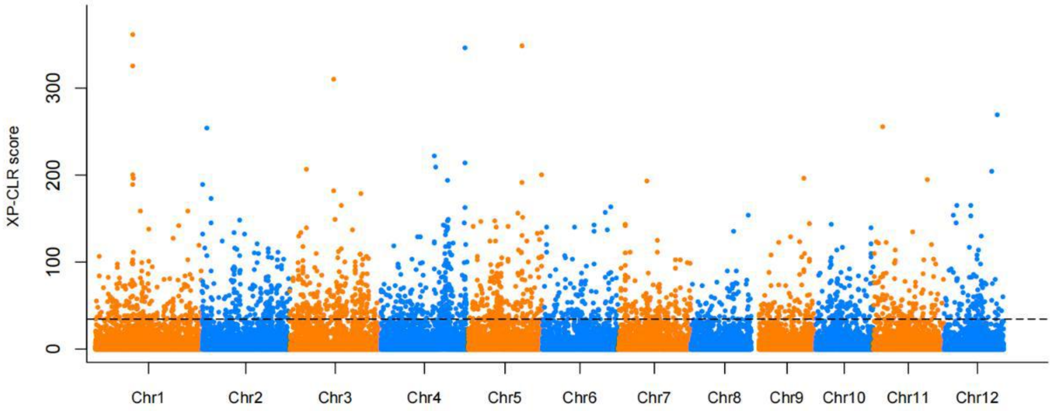

b.

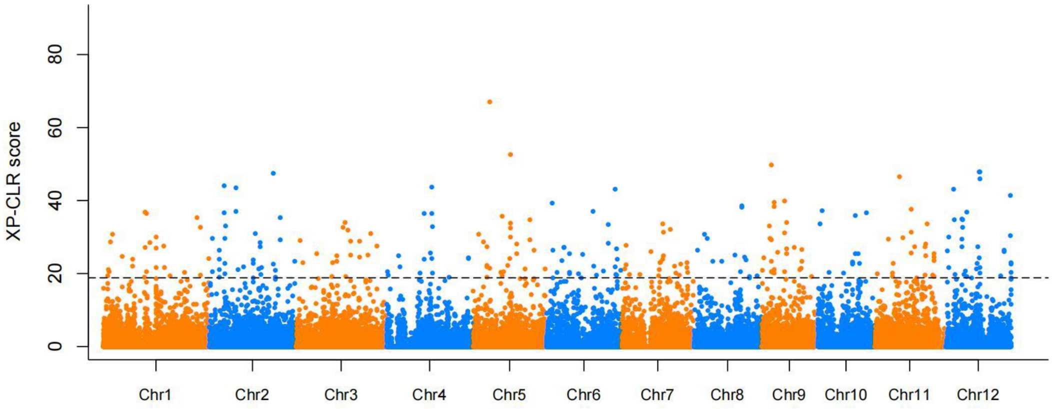

Figure. S11. (a) XP-CLR scores calculated by SNPs comparing wild rice with cultivated rice. (b) XP-CLR scores calculated by SVs comparing wild rice with cultivated rice.Chromosomes are painted with either blue or orange and the black line is defined using the top 5% XP-CLR scores.
