# Supplementary material for: Lost genome segments associate with trait diversity during rice domestication
Source: BMC Biol. 2023 Feb 1;21:20. doi: 10.1186/s12915-023-01512-6 (PMC9893545; doi:10.1186/s12915-023-01512-6)
Supplement: Supplementary file 2 — Additional file 2: Table S1. The results of 80 cultivated and wild rice genomes survey. Table S2. Summary of Nanopore Technologies sequencing data of JX1 and SL1 genome. Table S3. Summary of sequencing assembly data of JX1 and SL1 genome. Table S4. Details of the 12 pseudo-chromosomes of JX1 and SL1. Table S5. Summary of sequencing evaluation data of JX1 and SL1genome. Table S6. Repetitive elements in JX1 and SL1 genomes. Table S7. The information of full length transcriptome sequencing data of JX1 and SL1. Table S8. The transcriptome sequencing data from different tissues of JX1 and SL1 based on Illumina sequence. Table S9. Annotated predictions of gene structure. Table S10. Summary of the assembly of 13 cultivated and wild rice genomes. Table S11. The SV information for 13 cultivated and wild rice. Table S12. Sequence information for structural variation. Table S13. The annotation of SV. Table S14. The sample list for SV population analysis. Table S15. The significant association sites based on GWAS data. Table S16. 49 cloned genes overlapping with DELs (>10 kb) region. [file 12915_2023_1512_MOESM2_ESM.pdf]

Table S1: The results of 80 cultivated and wild rice genomes survey

Sample Name	Species name	Origin	Raw Data (Gb)	Q30(%)	The number of Kmer	Depth	GC content (%)	Genome size (Mb)	Estimated heterozygosity (%)
Z170	*O. rufipogon*	Laos	18.95	91.82	9,918,217,068	23	44	414.85	0
Z183	*O. rufipogon*	China	14.42	90.87	10,375,844,749	24	43	426.68	0
Z190	*O. rufipogon*	Philippine	24.59	91.50	15,698,362,585	25	43	613.19	0
Z218	*O. rufipogon*	China	12.52	88.19	9,748,484,915	22	44	424.82	0
Z37	*O. rufipogon*	Vietnam	14.32	92.56	13,744,912,226	25	44	547.12	0
Z225	*O. rufipogon*	China	12.46	89.37	16,983,389,331	39	44	435.46	0.32
JX4	*O. rufipogon*	China	19.56	91.84	10,075,846,740	23	44	423.21	0.58
JX2	*O. rufipogon*	China	11.65	89.40	11,047,399,786	25	44	426.56	0.6
JX5	*O. rufipogon*	China	18.09	91.89	10,504,532,205	24	44	421.59	0.6
JX6	*O. rufipogon*	China	19.24	91.84	10,909,872,023	24	44	433.12	0.6
JX3	*O. rufipogon*	China	12.31	88.27	11,587,367,245	26	43	422.01	0.7
JX11	*O. rufipogon*	China	11.59	88.80	12,029,225,360	27	44	426.14	0.74
Z33	*O. rufipogon*	China	13.88	89.59	14,262,516,769	33	44	423.63	0.8
Z9	*O. rufipogon*	China	13.53	92.46	13,399,978,155	31	45	424.11	0.82
SL1	*O. rufipogon*	Sri Lanka	18.83	91.54	15,003,292,397	36	44	412.69	0.9
Z166	*O. rufipogon*	Nepel	15.17	91.82	11,353,755,350	27	44	420.51	0.95
Z28	*O. rufipogon*	Nepel	13.81	89.47	17,576,053,086	42	44	413.38	0.95
Z111	*O. rufipogon*	Myanmar	17.92	91.50	9,833,970,758	23	43	418.85	1
Z116	*O. rufipogon*	Myanmar	16.48	91.41	10,520,140,780	25	43	420.81	1
Z21	*O. rufipogon*	China	12.92	88.82	14,178,450,250	34	44	410.17	1
Z26	*O. rufipogon*	China	14.02	89.33	14,489,619,475	35	43	408.18	1
Z5	*O. rufipogon*	China	14.38	92.17	11,774,540,069	28	44	420.52	1.01

Table S1 continued

Sample Name	Species name	Origin	Raw Data (Gb)	Q30(%)	The number of Kmer	Depth	GC content (%)	Genome size (Mb)	Estimated heterozygosity (%)
Z27	*O. rufipogon*	Laos	13.38	88.95	14,099,620,148	34	43	413.31	1.02
Z109	*O. rufipogon*	Myanmar	20.29	91.66	10,942,179,625	27	44	405.27	1.04
Z1	*O. rufipogon*	China	16.31	92.15	12,634,457,958	30	43	413.13	1.05
Z107	*O. rufipogon*	Myanmar	16.22	92.30	11,820,909,013	29	43	407.62	1.05
Z126	*O. rufipogon*	Myanmar	15.89	91.60	10,586,461,834	26	44	407.17	1.05
Z201	*O. rufipogon*	China	13.55	91.22	16,678,118,493	39	43	423.78	1.05
Z99	*O. rufipogon*	Sri Lanka	14.85	92.90	21,269,396,359	52	43	405.89	1.07
Z67	*O. rufipogon*	Sri Lanka	13.58	91.87	15,202,181,882	37	44	407.16	1.1
Z80	*O. rufipogon*	Sri Lanka	16.38	92.15	10,126,830,857	24	44	415.16	1.1
Z86	*O. rufipogon*	Sri Lanka	13.41	92.32	16,430,858,991	40	44	404.40	1.1
Z94	*O. rufipogon*	Sri Lanka	15.39	92.78	12,428,287,502	30	44	407.83	1.1
Z65	*O. rufipogon*	Sri Lanka	13.14	92.84	14,060,417,760	34	44	406.40	1.11
Z72	*O. rufipogon*	Sri Lanka	14.71	92.03	13,159,240,344	32	44	406.54	1.11
Z97	*O. rufipogon*	Sri Lanka	15.64	92.92	19,455,661,557	47	44	409.84	1.11
Z136	*O. rufipogon*	China	17.41	90.96	11,066,080,442	26	43	419.58	1.12
Z101	*O. rufipogon*	Sri Lanka	16.71	91.72	11,961,611,704	29	43	412.47	1.13
Z146	*O. rufipogon*	China	16.21	91.75	11,837,120,049	28	43	422.75	1.15
Z13	*O. rufipogon*	Nepel	17.63	91.53	16,306,741,861	39	43	412.41	1.16
Z162	*O. rufipogon*	Cambodia	17.49	92.08	11,714,327,024	28	43	418.37	1.18
Z186	*O. rufipogon*	Indonesia	22.44	91.78	16,975,261,911	41	43	409.98	1.27
Z29	*O. rufipogon*	Nepel	14.10	88.99	15,509,197,347	38	44	403.19	1.35
Z58	*O. rufipogon*	Sri Lanka	15.21	92.30	15,258,466,421	37	44	408.76	1.35

Table S1 continued

Sample Name	Species name	Origin	Raw Data (Gb)	Q30(%)	The number of Kmer	Depth	GC content (%)	Genome size (Mb)	Estimated heterozygosity (%)
JX1	*O. rufipogon*	China	11.96	88.64	11,299,978,328	26	43	424.93	0
JX10	*O. rufipogon*	China	11.53	88.52	12,121,421,842	27	44	425.22	0
JX12	*O. rufipogon*	China	12.96	89.84	11,530,357,028	25	44	437.09	0
JX7	*O. rufipogon*	China	19.59	91.85	12,343,903,672	28	44	421.38	0
JX8	*O. rufipogon*	China	14.70	91.23	10,261,186,363	23	43	429.80	0
JX9	*O. rufipogon*	China	15.58	91.72	10,351,794,152	23	44	438.93	0
D19031260	*O. sativa* ssp. *indica*	Philippines	22.34	93.45	16,871,140,824	40	44	371.78	0.16
D19031262	*O. sativa* ssp. *indica*	Vietnam	23.99	92.93	17,905,397,848	40	44	397.63	0.17
D19031263	*O. sativa* ssp. *indica*	Sri Lanka	33.26	93.00	24,547,055,320	56	44	388.34	0.29
D19031264	*O. sativa* ssp. *indica*	Thailand	21.78	92.44	17,442,985,824	38	44	409.03	0.14
D19031265	*O. sativa* ssp. *indica*	Vietnam	22.93	93.79	18,109,275,096	40	44	402.73	0.17
D19031266	*O. sativa* ssp. *indica*	Bangladesh	21.45	94.74	16,296,217,576	38	44	378.85	0.18
D19031267	*O. sativa* ssp. *indica*	China	17.80	91.83	14,299,505,552	30	43	426.65	0.26
D19031268	*O. sativa* ssp. *indica*	India	21.65	91.72	17,338,726,856	38	43	406.28	0.08
D19031269	*O. sativa* ssp. *indica*	Taiwan	20.40	94.09	16,127,461,192	38	44	374.41	0
D19031270	*O. sativa* ssp. *indica*	Malaysia	15.90	91.66	12,636,186,600	28	44	401.29	0
D19031271	*O. sativa* ssp. *indica*	India	19.19	91.38	15,383,549,472	34	44	402.46	0.06
D19031272	*O. sativa* ssp. *indica*	Laos	18.84	91.71	15,231,205,944	34	44	397.98	0
D19031273	*O. sativa* ssp. *indica*	Myanmar	13.41	91.48	10,826,082,708	22	44	442.09	0.15
D19031274	*O. sativa* ssp. *indica*	Indonesia	17.73	91.79	14,206,289,792	30	44	423.54	0.24
D19031275	*O. sativa* ssp. *indica*	Indonesia	21.07	91.51	16,918,146,416	36	43	419.95	0.25
D19031708	*O. sativa* ssp. *japonica*	Philippines	17.46	91.61	13,975,750,296	32	43	386.74	0.44

Table S1 continued

Sample Name	Species name	Origin	Raw Data (Gb)	Q30(%)	The number of Kmer	Depth	GC content (%)	Genome size (Mb)	Estimated heterozygosity (%)
D19031709	*O. sativa* ssp. *japonica*	China	23.33	91.84	18,531,677,400	42	43	391.23	0
D19031710	*O. sativa* ssp. *japonica*	Philippines	20.37	91.93	16,263,995,936	38	43	378	0.09
D19031711	*O. sativa* ssp. *japonica*	Japan	19.44	91.33	15,574,407,816	36	43	382.62	0
D19031712	*O. sativa* ssp. *japonica*	Bhutan	19.61	91.34	15,596,298,592	36	43	383.23	0
D19031713	*O. sativa* ssp. *japonica*	Indonesia	20.49	91.13	16,520,472,472	38	43	384.75	0.07
D19031714	*O. sativa* ssp. *japonica*	Japan	22.32	91.73	17,799,992,912	42	43	373.81	0
D19031715	*O. sativa* ssp. *japonica*	Bhutan	27.65	91.97	21,940,558,072	50	44	388.81	0
D19031716	*O. sativa* ssp. *japonica*	Japan	37.44	92.72	27,681,919,928	66	43	369.42	0
D19031717	*O. sativa* ssp. *japonica*	China	22.07	93.17	17,251,019,568	42	44	360.74	0
D19031718	*O. sativa* ssp. *japonica*	Philippines	21.91	92.31	17,215,676,800	40	43	380.39	0.19
D19031719	*O. sativa* ssp. *japonica*	Bhutan	19.61	91.91	15,584,287,904	36	44	382.9	0.13
D19031720	*O. sativa* ssp. *japonica*	Philippines	19.12	92.07	15,156,195,424	32	43	423.63	0.07
D19031722	*O. sativa* ssp. *japonica*	North Korea	19.92	91.34	15,972,481,616	36	44	393.68	0
D19031723	*O. sativa* ssp. *japonica*	Japan	14.17	92.06	11,225,729,400	26	44	381.76	0

Table S2: Summary of Nanopore Technologies sequencing data of JX1 and SL1 genome

	JX1	SL1
Cells	3	2
reads numbers	25,821,795,647	17,567,203,144
Total length (Gb)	25.8	17.6
Genome depth (X)	64.5	45.5
N50 size (bp)	52,256	52,317
Longest length (bp)	556,426	601,592
Mean length (bp)	33,874	30,209
Number of sequences	762,287	581,524

Table S3: Summary of sequencing assembly data of JX1 and SL1 genome

	**JX1**		**SL1**	
	Contig Length（bp）	Contig Number	Contig Length（bp）	Contig Number
**N50**	5825388	22	2665012	36
**N60**	4469890	30	1913227	53
**N70**	3394713	40	1436164	77
**N80**	2355652	55	954425	110
**N90**	1095364	81	669592	159
**Longest**	15735557	1	19511849	1
**Total**	**424926512**	**219**	**412733614**	**292**

Table S4: Details of the 12 pseudo-chromosomes of JX1 and SL1

Chromosome	JX1		SL1	
	Chromosome size	Contig number	Chromosome size	Contig number
1	46,942,102	21	45,668,546	18
2	37,398,581	13	37,158,464	17
3	40,051,614	12	38,333,721	14
4	37,854,111	16	37,368,780	32
5	30,569,654	10	29,024,140	15
6	33,048,860	14	32,006,640	22
7	31,388,483	18	32,342,471	26
8	28,646,020	8	29,715,933	26
9	25,692,589	9	25,697,743	15
10	25,937,495	18	24,292,433	20
11	34,387,699	33	33,919,688	39
12	29,301,304	14	27,877,648	27
Rates	98.65%		99.20%	
Total anchored	398,366,435		390,258,957	
Unassigned	5,451,543		3,147,250	
**Total**	**403,817,978**	**195**	**393,406,207**	**271**

Table S5: Summary of sequencing evaluation data of JX1 and SL1genome

	JX1		SL1	
	Number	Percent (%)	Number	Percent (%)
**Complete BUSCOs (C)**	1413	98.1	1388	96.4
**Complete and single-copy BUSCOs (S)**	1404	97.5	1334	92.6
**Complete and duplicated BUSCOs (D)**	9	0.6	54	3.8
**Fragmented BUSCOs (F)**	9	0.6	18	1.3
**Missing BUSCOs (M)**	18	1.3	34	2.3
**Total BUSCO groups searched**	1440	100	1440	100

Table S6: Repetitive elements in JX1 and SL1 genomes

Class	Order	Super family	JX1			SL1		
			Number of elements	Length of sequence (bp)	Percentage of sequence (%)	Number of elements	Length of sequence (bp)	Percentage of sequence (%)
**Class I**			**147976**	**115342918**	**28.18**	**140310**	**103171575**	**26.02**
	LTR		131192	109436943	26.74	123020	97074228	24.49
		Gypsy	84604	84846259	20.73	80265	73861782	18.63
		Unknown	26047	11767655	2.87	20321	11395676	2.87
		Copia	18828	11836483	2.89	21120	11269056	2.84
		Other	1713	986546	0.24	1314	547714	0.14
	LINE		13119	5422280	1.32	13570	5479975	1.38
		L1	13022	5414569	1.32	13458	5471464	1.38
		Other	97	7711	0	112	8511	0
	SINE		3665	483695	0.12	3720	617372	0.16
		Other	3665	483695	0.12	3720	617372	0.16
**Class II**			**342851**	**85365324**	**20.86**	**343260**	**83465007**	**21.05**
	DNA		283556	69796365	17.05	286875	68971324	17.4
		hAT-Ac	11798	3388329	0.83	11460	3239395	0.82
		MULE-MuDR	69998	18125116	4.43	70683	18272708	4.61
		TcMar-Stowaway	54070	8477545	2.07	58302	9073714	2.29
		PIF-Harbinger	62096	12169112	2.97	63367	12089639	3.05
		Unknown	27071	5896377	1.44	26582	5852470	1.48
		CMC-EnSpm	37152	17605364	4.3	36241	16409371	4.14
		hAT-Tip100	14429	2064958	0.5	13941	1981023	0.5

Table S6 continued

Class	Order	Super family	JX1 Number of elements	JX1 Length of sequence (bp)	JX1 Percentage of sequence (%)	SL1 Number of elements	SL1 Length of sequence (bp)	SL1 Percentage of sequence (%)
		hAT-Tag1	3113	574814	0.14	2592	522332	0.13
		MuLE-MuDR	1092	1077658	0.26	1076	1129203	0.28
		Other	2737	417092	0.1	2631	401469	0.1
	RC		59295	15568959	3.8	56385	14493683	3.66
		Helitron	59265	15567735	3.8	56350	14492248	3.66
		Other	30	1224	0	35	1435	0
**Total TEs**			**490827**	**200708242**	**49.03**	**483570**	**186636582**	**47.08**
Other			1881	1163171	0.28	2261	706674	0.18
Unknown			8835	2383285	0.58	10276	2998529	0.76
Simple repeats			1985	254991	0.06	1735	206921	0.05
Low complexity			17	2167	0	16	2154	0
**Total Repeats**			**503545**	**204511856**	**49.96**	**497858**	**190550860**	**48.07**

Table S7: The information of full length transcriptome sequencing data of JX1 and SL1

Sample Name	Total number of Bases	Total number of reads	Mean length of reads	The N50 length of reads	Number of full-length reads	Total mapped rate
JX1	17743636341	486870	36444	80250	422,077(87.98%)	99.73%
SL1	22693827394	581101	39053	72144	505,848(87.05%)	98.49%

Table S8: The transcriptome sequencing data from different tissues of JX1 and SL1 based on illumina sequence.

	JX1					SL1				
**Tissue Description**	**Flower**	**Panicle**	**Stem**	**Leaf**	**Root**	**Flower**	**Panicle**	**Stem**	**Leaf**	**Root**
**Raw reads**	55,022,268	55,259,147	84,375,320	37,370,298	42,313,195	55,113,670	38,248,415	38,509,074	54,532,538	39,469,686
**Raw_bases (Gb)**	16.53	11.47	11.55	16.36	11.84	16.51	16.58	25.31	11.21	12.69
**Clean_reads**	53,818,606	37,114,770	37,601,131	53,705,649	39,414,015	53,695,093	54,433,717	83,011,128	36,449,313	41,540,559
**Clean_bases (Gb)**	16.15	11.13	11.28	16.11	11.82	16.11	16.33	24.90	10.93	12.46
**Average Q30 (%)**	94	94	94	94	95	94	94	94	94	95
**Average GC (%)**	54	53	53	53	51	52	55	53	53	53
**Total reads**	116,814,720	116,204,286	175,366,597	78,159,411	87,574,723	114,412,544	78,302,117	80,353,133	116,310,073	83,718,401
**Mapped reads**	111,499,607	110,699,493	167,645,934	74,358,563	82,752,184	105,782,574	71,856,764	73,789,535	107,917,850	72,732,194
**Mapped rates (%)**	0.95	0.95	0.96	0.95	0.94	0.92	0.92	0.92	0.93	0.87
**Index sequence**	AGTCCTC-AGTCCTC	CGCCTCA-CGCCTCA	TCATGGT-TCATGGT	GTTACCG-GTTACCG	AGAATTA+AGAATTA	TGGCAAT-TGGCAAT	AACGTTA-AACGTTA	AAGATTC-AAGATTC	GTACCGG-GTACCGG	TTCTAGG-TTCTAGG

Table S9: Annotated predictions of gene structure

Gene set		Sample	Total number of gene	Average transcript length (bp)	Average CDS length (bp)	Average exons numberper gene	Average exon length (bp)	Average intron length (bp)
**De novo**	**Augustus**	**JX1**	39,321	2,761.68	1,016.78	4.7	216.48	471.98
	**Snap**		56,136	3,985.91	756.47	3.67	206.1	1,209.34
**Homology**	**Arabidopsis**		29,625	1,974.23	895.58	3.55	252.43	423.38
	**Hordeum**		40,767	1,761.85	824.81	3.23	255.7	421.03
	**Oryza.sativa**		97,326	1,019.75	525.82	2.52	208.67	325
	**Physcomitrella**		25,458	1,866.16	821.55	3.26	252.25	462.86
	**Zea.mays**		32,649	2,242.57	980.66	3.79	258.7	452.21
	**Triticum**		33,696	2,381.57	1,014.34	4.14	244.78	434.9
**Transcription**	**PASA**		47,279	2,760.57	1,143.08	4.71	242.74	436.1
**Final set**	**EVM**		39,252	2,910.48	1,045.93	4.54	230.53	527.15
**De novo**	**Augustus**	**SL1**	39,385	2,669.67	975.99	4.66	209.54	463.03
	**Snap**		57,218	3,978.73	742.76	3.72	199.91	1,191.68
**Homology**	**Arabidopsis**		27,938	2,039.48	906.33	3.78	239.98	408.11
	**Hordeum**		38,255	1,811.39	840.24	3.49	240.72	389.97
	**Oryza.sativa**		85,849	1,078.06	547.74	2.79	196.55	296.82
	**Physcomitrella**		24,098	1,810.25	820.59	3.43	239.31	407.49
	**Zea.mays**		31,018	2,313.81	998.22	4.09	244.19	426.1
	**Triticum**		32,301	2,369.25	1,010.12	4.39	230.21	401.19
**Transcription**	**PASA**		44,636	2,683.16	1,113.35	4.66	238.86	428.78
**Final set**	**EVM**		39,343	2,834.30	1,000.37	4.51	221.74	522.27

Table S10: Summary of the assembly of 13 cultivated and wild rice genomes.

Sample	Species	GenBank accession	Genome size (Mb)	The length of contig N50(Mb)
W-JX1	*O. rufipogon*	in this study	409.3	5.83
W-SL1	*O. rufipogon*	in this study	396.4	2.67
W-CAS	*O. rufipogon*	PRJCA002346	380.5	1.09
W-GLZ	*O. rufipogon*	PRJCA002385	399.8	13.2
Nipponbare	*O. sativa* ssp. *japonica*	GCA_003865235.1	379.6	16.97
J-SN265	*O. sativa* ssp. *japonica*	GCA_003449065.1	378.1	7.16
J-SJ18	*O. sativa* ssp. *japonica*	GCA_002573525.1	418.9	2.52
I-9311	*O. sativa* ssp. *indica*	GCA_003865215.1	395.4	9.64
I-IR8	*O. sativa* ssp. *indica*	GCA_001889745.1	389.1	14.56
I-MH63	*O. sativa* ssp. *indica*	GCA_001623365.2	387.4	27.02
I-N22	*O. sativa* ssp. *indica*	GCA_001952365.1	362.3	0.95
I-R498	*O. sativa* ssp. *indica*	GCA_002151415.1	391.0	25.58
I-ZS97	*O. sativa* ssp. *indica*	GCA_001623345.2	387.3	25.68

Table S11: the SV information for 13 cultivated and wild rice

	Insertion			Deletion			Total
	**Length**	**The longest**	**Count**	**Length**	**The longest**	**Count**	**Length**
I-9311	4E+07	91914	35051	3.6E+07	94822	25662	7.6E+07
I-IR8	4.3E+07	95802	36329	3.7E+07	94822	27151	8E+07
I-MH63	4E+07	91944	32710	3.4E+07	99788	23792	7.4E+07
I-N22	1.8E+07	90123	21279	2.3E+07	97821	16414	4.1E+07
I-R498	4.3E+07	96539	36196	3.6E+07	99325	25938	7.9E+07
I-ZS97	3.8E+07	88584	32605	3.4E+07	99076	23930	7.2E+07
J-SJ18	9077568	83331	9712	9278615	91946	7160	1.8E+07
J-SN265	9989507	87451	8707	8578196	97240	6722	1.9E+07
W-CAS	4.3E+07	96896	34060	3.4E+07	95435	25047	7.6E+07
W-GLZ	4.6E+07	97749	47397	4.8E+07	94590	34827	9.4E+07
W-JX1	4E+07	99420	32963	3E+07	94595	27993	7E+07
W-SL1	3.8E+07	98501	37220	3.5E+07	94595	35825	7.4E+07

Table S12: Sequence information for structural variation.

	Primer sequence F	Primer sequence R	Start location	End location	Sequence Length
Contig45.379	CCCAACGGCTTCGATTTCTT	AGCCATGTTCATATTCTTGCG	7	753	746
Contig10.00	GAGGAAGCTGAGGCTGAAGAA	GGTGAAGCGCAGTCTTGAGA	105	548	443
Contig29.560	AGTGCGTACAACAACGAAAAGG	AGCTCCTCATGGGTCTTGTAAAC	139	917	778
Contig44.102	CCTCCCTCAACTCAGGTCAG	GTCAAAATCCCTCGACTGTAAGA	15	1112	1097
Contig44.164	CTATGCTCTCCCACCTTCCTT	CTATGCTCTCCCACCTTCCTT	154	998	844
Contig29.167	GAAGACGAAGAAACTAGTCAGGGTG	TACATCGTAGCCAGTTGGAATAATT	6	672	666
Contig12.663	CACTACCGGACGCTGGGAAT	ATCAATAATTTGCCTGCCGTCT	25	522	497
Contig29.32	GGAGGACAAGACGGTGATCAT	CTGAACTTGTTGGCACCCTT	3	815	812
Contig6.1257	CTGTGACGGACGAGGACTTC	GAGGTAAGACGGGTCAAGAATT	143	1097	954
Contig10.143	CCTCTCATCTTCATGGACGC	CTCTCTGGACAGGGTGAAGG	109	750	641

Table S13: The annotation of SV

Category		SV number	Sum
gene region	exonic	31969	58007
	intronic	26038	
regulation region	downstream	23680	59059
	upstream	27647	
	upstream;downstream	3321	
	UTR3	2869	
	UTR5	1542	
intergenic	intergenic	211602	211602

Table S14: The sample list for SV population analysis.

Sample name	Origin	Cluster	Depth	Q30(%)
DNA103	China	*O. rufipogon*	25	94.05
DNA104	China	*O. rufipogon*	21	94.69
DNA105	China	*O. rufipogon*	21	93.77
DNA106	China	*O. rufipogon*	23	94.53
DNA107	China	*O. rufipogon*	26	94.56
DNA108	China	*O. rufipogon*	25	94.62
DNA110	China	*O. rufipogon*	16	92.05
DNA111	China	*O. rufipogon*	28	92.54
DNA112	China	*O. rufipogon*	21	91.58
DNA113	China	*O. rufipogon*	23	94.35
DNA114	China	*O. rufipogon*	23	94.44
DNA115	China	*O. rufipogon*	30	92.94
DNA116	China	*O. rufipogon*	19	92.74
DNA118	China	*O. rufipogon*	23	93.36
DNA119	China	*O. rufipogon*	20	91.66
DNA120	China	*O. rufipogon*	30	93.65
DNA121	China	*O. rufipogon*	39	93.28
DNA122	China	*O. rufipogon*	27	93.46
DNA123	China	*O. rufipogon*	26	92.63
DNA124	China	*O. rufipogon*	34	93.19
DNA125	China	*O. rufipogon*	25	93.12
DNA126	China	*O. rufipogon*	25	93.10
DNA127	China	*O. rufipogon*	19	92.47
DNA128	China	*O. rufipogon*	22	91.68
DNA129	China	*O. rufipogon*	28	92.71
DNA130	China	*O. rufipogon*	20	91.12
DNA131	China	*O. rufipogon*	40	92.74
DNA132	China	*O. rufipogon*	22	92.15
DNA133	China	*O. rufipogon*	42	93.45
DNA134	China	*O. rufipogon*	31	93.96
DNA137	China	*O. rufipogon*	21	92.41
DNA138	China	*O. rufipogon*	23	93.62
DNA139	China	*O. rufipogon*	30	90.95
DNA140	China	*O. rufipogon*	23	92.55
DNA141	China	*O. rufipogon*	20	91.96
DNA142	China	*O. rufipogon*	23	93.73
R.guad33	China	*O. rufipogon*	9	85.48
R.guad36	China	*O. rufipogon*	8	91.74
R.guad40	China	*O. rufipogon*	9	86.77
R.guad41	China	*O. rufipogon*	8	89.11
R.guad43	China	*O. rufipogon*	8	91.48
R.guad44	China	*O. rufipogon*	8	91.43

Table S14 continued

Sample name	Origin	Cluster	Depth	Q30(%)
R.guad49	China	*O. rufipogon*	8	86.53
R.guax41	China	*O. rufipogon*	9	90.45
R.guax42	China	*O. rufipogon*	9	89.68
R.guax43	China	*O. rufipogon*	9	89.67
R.guax47	China	*O. rufipogon*	8	89.76
R.guax49	China	*O. rufipogon*	8	80.10
R.guax50	China	*O. rufipogon*	10	90.13
R.huna53	China	*O. rufipogon*	8	84.70
R.huna54	China	*O. rufipogon*	9	89.08
R.huna56	China	*O. rufipogon*	9	89.25
R.huna57	China	*O. rufipogon*	8	84.71
R.huna58	China	*O. rufipogon*	10	90.04
R.huna59	China	*O. rufipogon*	8	91.19
R.jiax01	China	*O. rufipogon*	8	90.45
R.jiax02	China	*O. rufipogon*	8	90.03
R.jiax04	China	*O. rufipogon*	8	89.69
R.sril20	Sri Lanka	*O. rufipogon*	8	89.45
R.thai10	Thailand	*O. rufipogon*	9	89.02
DNA109	China	*O. rufipogon*	30	94.59
DNA135	China	*O. rufipogon*	22	92.89
DNA136	China	*O. rufipogon*	25	93.74
N.indi14	India	*O. rufipogon*	8	89.93
N.myan01	Myanmar	*O. rufipogon*	8	87.82
N.myan03	Myanmar	*O. rufipogon*	10	89.42
N.myan05	Myanmar	*O. rufipogon*	8	89.12
N.nepa04	Nepal	*O. rufipogon*	7	85.44
N.nepa05	Nepal	*O. rufipogon*	9	89.76
N.nepa07	Nepal	*O. rufipogon*	8	91.06
N.sril12	Sri Lanka	*O. rufipogon*	9	86.71
N.sril13	Sri Lanka	*O. rufipogon*	10	86.42
N.sril14	Sri Lanka	*O. rufipogon*	8	88.19
N.sril15	Sri Lanka	*O. rufipogon*	8	86.86
N.sril16	Sri Lanka	*O. rufipogon*	9	86.52
R.camb04	Cambodia	*O. rufipogon*	8	84.62
R.camb05	Cambodia	*O. rufipogon*	8	85.85
R.fuji22	China	*O. rufipogon*	9	83.83
R.guad30	China	*O. rufipogon*	8	88.36
R.hain48	China	*O. rufipogon*	8	90.10
R.hain51	China	*O. rufipogon*	8	87.07
R.hain52	China	*O. rufipogon*	8	86.74
R.hain53	China	*O. rufipogon*	10	90.74
R.hain55	China	*O. rufipogon*	10	88.36

Table S14 continued

Sample name	Origin	Cluster	Depth	Q30(%)
R.hain57	China	*O. rufipogon*	9	88.01
R.hain58	China	*O. rufipogon*	9	84.49
R.hain60	China	*O. rufipogon*	8	90.81
R.indi12	India	*O. rufipogon*	8	88.20
R.indo11	Indonesia	*O. rufipogon*	9	88.38
R.indo12	Indonesia	*O. rufipogon*	12	88.80
R.laos15	Laos	*O. rufipogon*	8	86.23
R.laos16	Laos	*O. rufipogon*	8	86.28
R.myan03	Myanmar	*O. rufipogon*	8	90.90
R.myan06	Myanmar	*O. rufipogon*	8	88.94
R.nepa20	Nepal	*O. rufipogon*	8	89.44
R.nepa22	Nepal	*O. rufipogon*	8	82.42
R.nepa23	Nepal	*O. rufipogon*	8	86.89
R.nepa25	Nepal	*O. rufipogon*	8	87.18
R.png01	PNG	*O. rufipogon*	8	88.59
R.png03	PNG	*O. rufipogon*	9	86.46
R.png04	PNG	*O. rufipogon*	9	88.39
R.thai20	Thailand	*O. rufipogon*	6	90.97
R.viet31	Vietnam	*O. rufipogon*	7	84.58
R.viet32	Vietnam	*O. rufipogon*	8	83.96
R.viet35	Vietnam	*O. rufipogon*	7	84.41
R.yuna03	China	*O. rufipogon*	8	82.61
R.yuna04	China	*O. rufipogon*	12	87.53
R.yuna05	China	*O. rufipogon*	8	85.45
DNA101	China	*O. rufipogon*	24	94.43
DNA102	China	*O. rufipogon*	27	94.92
DNA147	China	*O. rufipogon*	30	93.67
N.camb03	Cambodia	*O. rufipogon*	9	84.88
N.indi05	India	*O. rufipogon*	9	86.94
N.indi07	India	*O. rufipogon*	8	88.85
N.indi08	India	*O. rufipogon*	8	91.80
N.indi10	India	*O. rufipogon*	7	91.43
N.indi11	India	*O. rufipogon*	7	88.74
DNA117	China	*O. rufipogon*	25	93.54
DNA150	China	*O. rufipogon*	22	94.63
Z170	Laos	*O. rufipogon*	23	91.82
Z183	China	*O. rufipogon*	24	90.87
Z190	Philippine	*O. rufipogon*	25	91.50
Z218	China	*O. rufipogon*	22	88.19
Z37	Vietnam	*O. rufipogon*	25	92.56
Z225	China	*O. rufipogon*	39	89.37
JX4	China	*O. rufipogon*	23	91.84

Table S14 continued

Sample name	Origin	Cluster	Depth	Q30(%)
JX2	China	*O. rufipogon*	25	89.40
JX5	China	*O. rufipogon*	24	91.89
JX6	China	*O. rufipogon*	24	91.84
JX3	China	*O. rufipogon*	26	88.27
JX11	China	*O. rufipogon*	27	88.80
Z33	China	*O. rufipogon*	33	89.59
Z9	China	*O. rufipogon*	31	92.46
SL1	Sri Lanka	*O. rufipogon*	36	91.54
Z166	Nepel	*O. rufipogon*	27	91.82
Z28	Nepel	*O. rufipogon*	42	89.47
Z111	Myanmar	*O. rufipogon*	23	91.50
Z116	Myanmar	*O. rufipogon*	25	91.41
Z21	China	*O. rufipogon*	34	88.82
Z26	China	*O. rufipogon*	35	89.33
Z5	China	*O. rufipogon*	28	92.17
Z27	Laos	*O. rufipogon*	34	88.95
Z109	Myanmar	*O. rufipogon*	27	91.66
Z1	China	*O. rufipogon*	30	92.15
Z107	Myanmar	*O. rufipogon*	29	92.30
Z126	Myanmar	*O. rufipogon*	26	91.60
Z201	China	*O. rufipogon*	39	91.22
Z99	Sri Lanka	*O. rufipogon*	52	92.90
Z67	Sri Lanka	*O. rufipogon*	37	91.87
Z80	Sri Lanka	*O. rufipogon*	24	92.15
Z86	Sri Lanka	*O. rufipogon*	40	92.32
Z94	Sri Lanka	*O. rufipogon*	30	92.78
Z65	Sri Lanka	*O. rufipogon*	34	92.84
Z72	Sri Lanka	*O. rufipogon*	32	92.03
Z97	Sri Lanka	*O. rufipogon*	47	92.92
Z136	China	*O. rufipogon*	26	90.96
Z101	Sri Lanka	*O. rufipogon*	29	91.72
Z146	China	*O. rufipogon*	28	91.75
Z13	Nepel	*O. rufipogon*	39	91.53
Z162	Cambodia	*O. rufipogon*	28	92.08
Z186	Indonesia	*O. rufipogon*	41	91.78
Z29	Nepel	*O. rufipogon*	38	88.99
Z58	Sri Lanka	*O. rufipogon*	37	92.30
JX1	China	*O. rufipogon*	26	88.64
JX10	China	*O. rufipogon*	27	88.52
JX12	China	*O. rufipogon*	25	89.84
JX7	China	*O. rufipogon*	28	91.85
JX8	China	*O. rufipogon*	23	91.23

Table S14 continued

Sample name	Origin	Cluster	Depth	Q30(%)
JX9	China	*O. rufipogon*	23	91.72
272_I8986	India	*O. sativa ssp. indica*	33	96.60
287_I11445	India	*O. sativa ssp. indica*	27	96.64
289_I9313	India	*O. sativa ssp. indica*	28	96.80
326_B8509	Bangladesh	*O. sativa ssp. indica*	26	93.57
329_I8957	India	*O. sativa ssp. indica*	26	92.95
335_S8699	Sri Lanka	*O. sativa ssp. indica*	31	93.34
340_I8530	India	*O. sativa ssp. indica*	23	95.32
344_I11490	India	*O. sativa ssp. indica*	37	93.11
373_N11561	Nepal	*O. sativa ssp. indica*	26	95.76
374_V9637	Vietnam	*O. sativa ssp. indica*	29	92.83
375_A9767	UnitedStates	*O. sativa ssp. indica*	25	93.01
396_V8996	Vietnam	*O. sativa ssp. indica*	32	92.94
399_I9557	India	*O. sativa ssp. indica*	25	95.33
410_I8450	India	*O. sativa ssp. indica*	25	95.13
412_I11448	India	*O. sativa ssp. indica*	25	95.32
B006	Vietnam	*O. sativa ssp. indica*	17	92.59
B073	China	*O. sativa ssp. indica*	22	92.02
DNA11	Malaysia	*O. sativa ssp. indica*	18	91.91
DNA12	India	*O. sativa ssp. indica*	22	91.66
DNA13	Laos	*O. sativa ssp. indica*	21	91.97
DNA14	Myanmar	*O. sativa ssp. indica*	20	91.25
DNA15	Indonesia	*O. sativa ssp. indica*	20	92.09
DNA16	Indonesia	*O. sativa ssp. indica*	24	91.80
DNA18	Bangladesh	*O. sativa ssp. indica*	23	94.35
DNA19	India	*O. sativa ssp. indica*	21	94.12
DNA21	Bangladesh	*O. sativa ssp. indica*	19	94.01
DNA25	Vietnam	*O. sativa ssp. indica*	20	92.21
DNA26	Bangladesh	*O. sativa ssp. indica*	23	95.05
DNA3	Vietnam	*O. sativa ssp. indica*	27	93.28
DNA30	Indonesia	*O. sativa ssp. indica*	20	91.93
DNA31	Bangladesh	*O. sativa ssp. indica*	23	94.73
DNA36	Indonesia	*O. sativa ssp. indica*	30	94.73
DNA38	Laos	*O. sativa ssp. indica*	22	91.16
DNA39	Myanmar	*O. sativa ssp. indica*	19	92.27
DNA4	Sri Lanka	*O. sativa ssp. indica*	37	93.34
DNA44	China	*O. sativa ssp. indica*	26	92.53
DNA45	Indonesia	*O. sativa ssp. indica*	18	92.08
DNA46	Laos	*O. sativa ssp. indica*	22	92.15
DNA5	Thailand	*O. sativa ssp. indica*	25	92.69
DNA6	Vietnam	*O. sativa ssp. indica*	26	94.06
DNA7	Bangladesh	*O. sativa ssp. indica*	24	95.01

Table S14 continued

Sample name	Origin	Cluster	Depth	Q30(%)
DNA8	China	*O. sativa ssp. indica*	20	92.09
DNA85	Philippines	*O. sativa ssp. indica*	22	92.68
DNA92	China	*O. sativa ssp. indica*	17	92.47
DNA98	China	*O. sativa ssp. indica*	26	94.52
S10151	Thailand	*O. sativa ssp. indica*	35	93.42
S10177	China	*O. sativa ssp. indica*	33	90.81
S10547	Myanmar	*O. sativa ssp. indica*	14	91.95
S10681	Laos	*O. sativa ssp. indica*	14	93.67
S10682	Laos	*O. sativa ssp. indica*	17	93.65
S10762	Indonesia	*O. sativa ssp. indica*	17	91.24
S10835	India	*O. sativa ssp. indica*	14	92.10
S10942	Indonesia	*O. sativa ssp. indica*	15	93.75
S11076	Laos	*O. sativa ssp. indica*	12	94.02
S11301	India	*O. sativa ssp. indica*	15	92.98
S11318	Indonesia	*O. sativa ssp. indica*	11	92.86
S11351	India	*O. sativa ssp. indica*	16	92.21
S11358	India	*O. sativa ssp. indica*	13	92.00
S11416	India	*O. sativa ssp. indica*	19	89.95
S11517	Philippines	*O. sativa ssp. indica*	38	93.09
S11543	Myanmar	*O. sativa ssp. indica*	13	91.96
S11558	Bangladesh	*O. sativa ssp. indica*	37	94.79
S11645	India	*O. sativa ssp. indica*	34	92.07
S11648	India	*O. sativa ssp. indica*	15	91.26
S11678	Thailand	*O. sativa ssp. indica*	39	95.11
S11682	Thailand	*O. sativa ssp. indica*	39	93.31
S11685	Thailand	*O. sativa ssp. indica*	30	92.52
S11693	China	*O. sativa ssp. indica*	50	95.41
S11700	Thailand	*O. sativa ssp. indica*	30	92.61
S11705	Thailand	*O. sativa ssp. indica*	28	92.21
S11719	Thailand	*O. sativa ssp. indica*	28	92.37
S11721	Thailand	*O. sativa ssp. indica*	27	92.08
S11744	China	*O. sativa ssp. indica*	30	93.10
S11748	China	*O. sativa ssp. indica*	27	92.89
S11820	Myanmar	*O. sativa ssp. indica*	26	91.47
S11823	India	*O. sativa ssp. indica*	28	90.95
S11902	Indonesia	*O. sativa ssp. indica*	15	91.69
S11918	India	*O. sativa ssp. indica*	13	93.04
S12121	Laos	*O. sativa ssp. indica*	18	92.48
S12127	Laos	*O. sativa ssp. indica*	18	92.07
S12128	Laos	*O. sativa ssp. indica*	13	92.49
S12193	Laos	*O. sativa ssp. indica*	13	90.01
S12221	Laos	*O. sativa ssp. indica*	13	93.81

Table S14 continued

Sample name	Origin	Cluster	Depth	Q30(%)
S12225	Laos	*O. sativa ssp. indica*	13	94.02
S12287	Myanmar	*O. sativa ssp. indica*	14	94.23
S12302	Laos	*O. sativa ssp. indica*	13	93.86
S12305	Laos	*O. sativa ssp. indica*	14	94.36
S12334	Laos	*O. sativa ssp. indica*	15	89.83
S8492	Malaysia	*O. sativa ssp. indica*	33	93.31
S8568	India	*O. sativa ssp. indica*	44	95.21
S8679	Thailand	*O. sativa ssp. indica*	33	92.53
S8703	Bangladesh	*O. sativa ssp. indica*	27	93.88
S8725	Indonesia	*O. sativa ssp. indica*	21	93.29
S8737	Bangladesh	*O. sativa ssp. indica*	37	95.03
S8754	India	*O. sativa ssp. indica*	37	92.43
S8956	Indonesia	*O. sativa ssp. indica*	37	94.58
S8980	Thailand	*O. sativa ssp. indica*	33	92.60
S9006	Thailand	*O. sativa ssp. indica*	35	94.50
S9019	Thailand	*O. sativa ssp. indica*	36	92.58
S9072	Bangladesh	*O. sativa ssp. indica*	35	93.69
S9139	Bangladesh	*O. sativa ssp. indica*	37	92.97
S9174	Bangladesh	*O. sativa ssp. indica*	35	93.19
S9188	Indonesia	*O. sativa ssp. indica*	32	93.03
S9209	Thailand	*O. sativa ssp. indica*	32	93.91
S9218	Bangladesh	*O. sativa ssp. indica*	32	93.50
S9317	Vietnam	*O. sativa ssp. indica*	39	95.11
S9594	Bangladesh	*O. sativa ssp. indica*	42	95.26
S9606	Bangladesh	*O. sativa ssp. indica*	33	93.54
S9936	Sri Lanka	*O. sativa ssp. indica*	14	93.11
B164	China	*O. sativa ssp. indica*	17	92.62
S10534	India	*O. sativa ssp. indica*	15	92.14
S10602	Bangladesh	*O. sativa ssp. indica*	17	95.05
S10603	Bangladesh	*O. sativa ssp. indica*	23	91.46
S10605	Bangladesh	*O. sativa ssp. indica*	20	90.49
S10608	India	*O. sativa ssp. indica*	12	94.92
S10623	Nepal	*O. sativa ssp. indica*	14	93.30
S10735	Nepal	*O. sativa ssp. indica*	10	90.22
S10736	Nepal	*O. sativa ssp. indica*	11	90.76
S10861	India	*O. sativa ssp. indica*	24	91.12
S10871	India	*O. sativa ssp. indica*	20	90.12
S10876	India	*O. sativa ssp. indica*	12	92.29
S10891	India	*O. sativa ssp. indica*	14	92.28
S10892	India	*O. sativa ssp. indica*	24	90.81
S10927	Nepal	*O. sativa ssp. indica*	16	93.51
S10930	Bangladesh	*O. sativa ssp. indica*	14	93.61

Table S14 continued

Sample name	Origin	Cluster	Depth	Q30(%)
S10963	Bangladesh	*O. sativa ssp. indica*	12	92.83
S10965	Bangladesh	*O. sativa ssp. indica*	14	92.92
S11015	Bangladesh	*O. sativa ssp. indica*	12	94.03
S11016	Bangladesh	*O. sativa ssp. indica*	15	94.22
S11027	Pakistan	*O. sativa ssp. indica*	20	92.07
S11034	Pakistan	*O. sativa ssp. indica*	14	94.16
S11037	Pakistan	*O. sativa ssp. indica*	20	92.01
S11047	Bangladesh	*O. sativa ssp. indica*	21	90.45
S11048	Bangladesh	*O. sativa ssp. indica*	21	90.12
S11049	Bangladesh	*O. sativa ssp. indica*	14	89.06
S11050	Bangladesh	*O. sativa ssp. indica*	20	90.55
S11051	Bangladesh	*O. sativa ssp. indica*	20	90.46
S11052	Bangladesh	*O. sativa ssp. indica*	25	89.85
S11053	Bangladesh	*O. sativa ssp. indica*	25	90.43
S11054	Bangladesh	*O. sativa ssp. indica*	16	89.86
S11055	Bangladesh	*O. sativa ssp. indica*	17	89.72
S11056	Bangladesh	*O. sativa ssp. indica*	20	90.29
S11057	Bangladesh	*O. sativa ssp. indica*	23	90.44
S11058	Bangladesh	*O. sativa ssp. indica*	26	90.58
S11059	Bangladesh	*O. sativa ssp. indica*	13	90.36
S11272	India	*O. sativa ssp. indica*	12	92.44
S11274	India	*O. sativa ssp. indica*	15	92.86
S11277	India	*O. sativa ssp. indica*	22	90.58
S11298	India	*O. sativa ssp. indica*	27	90.92
S11324	Bangladesh	*O. sativa ssp. indica*	14	94.47
S11454	India	*O. sativa ssp. indica*	11	94.25
S11456	India	*O. sativa ssp. indica*	11	93.48
S11481	Bangladesh	*O. sativa ssp. indica*	31	95.24
S11482	Bangladesh	*O. sativa ssp. indica*	33	93.64
S11483	Bangladesh	*O. sativa ssp. indica*	36	95.24
S11602	India	*O. sativa ssp. indica*	15	94.15
S11603	India	*O. sativa ssp. indica*	11	93.25
S11617	India	*O. sativa ssp. indica*	38	95.53
S11618	India	*O. sativa ssp. indica*	41	94.94
S11619	India	*O. sativa ssp. indica*	35	95.17
S11917	India	*O. sativa ssp. indica*	11	92.85
S11963	India	*O. sativa ssp. indica*	13	93.49
S8398	Pakistan	*O. sativa ssp. indica*	10	92.76
S8554	India	*O. sativa ssp. indica*	12	93.29
S8641	Bangladesh	*O. sativa ssp. indica*	15	93.57
S8721	Bangladesh	*O. sativa ssp. indica*	13	93.45
S9283	Pakistan	*O. sativa ssp. indica*	10	93.21

Table S14 continued

Sample name	Origin	Cluster	Depth	Q30(%)
S9449	Pakistan	*O. sativa ssp. indica*	38	94.80
S9610	India	*O. sativa ssp. indica*	11	93.85
S9626	Bangladesh	*O. sativa ssp. indica*	14	93.48
188_P10361	Philippines	*O. sativa ssp. indica*	26	95.50
193_I10148	India	*O. sativa ssp. indica*	27	95.77
198_P10167	Philippines	*O. sativa ssp. indica*	28	95.87
213_V11507	Vietnam	*O. sativa ssp. indica*	24	95.16
219_P10234	Philippines	*O. sativa ssp. indica*	31	95.94
237_P10394	Philippines	*O. sativa ssp. indica*	25	95.45
278_B8930	Bangladesh	*O. sativa ssp. indica*	35	95.21
294_P10375	Philippines	*O. sativa ssp. indica*	27	96.87
353_S9968	Sri Lanka	*O. sativa ssp. indica*	24	91.52
B010	Malaysia	*O. sativa ssp. indica*	17	92.59
B061	China	*O. sativa ssp. indica*	19	92.03
B094	China	*O. sativa ssp. indica*	19	92.62
B208	China	*O. sativa ssp. indica*	28	92.72
B210	China	*O. sativa ssp. indica*	25	92.58
B217	China	*O. sativa ssp. indica*	18	92.32
CX10	China	*O. sativa ssp. indica*	22	91.31
DNA1	Philippines	*O. sativa ssp. indica*	25	93.70
DNA10	China	*O. sativa ssp. indica*	23	94.34
DNA2	Philippines	*O. sativa ssp. indica*	22	91.37
DNA20	Sri Lanka	*O. sativa ssp. indica*	30	91.61
DNA22	China	*O. sativa ssp. indica*	24	91.54
DNA23	Thailand	*O. sativa ssp. indica*	20	92.66
DNA24	Sri Lanka	*O. sativa ssp. indica*	22	91.66
DNA27	China	*O. sativa ssp. indica*	20	92.36
DNA28	India	*O. sativa ssp. indica*	30	94.77
DNA29	India	*O. sativa ssp. indica*	21	92.09
DNA32	China	*O. sativa ssp. indica*	32	93.96
DNA33	Nepal	*O. sativa ssp. indica*	31	91.99
DNA34	Philippines	*O. sativa ssp. indica*	24	94.97
DNA35	Laos	*O. sativa ssp. indica*	24	92.48
DNA37	China	*O. sativa ssp. indica*	23	94.54
DNA41	China	*O. sativa ssp. indica*	19	92.69
DNA42	China	*O. sativa ssp. indica*	19	91.72
DNA43	China	*O. sativa ssp. indica*	20	92.46
DNA47	China	*O. sativa ssp. indica*	24	92.03
DNA48	China	*O. sativa ssp. indica*	25	92.07
DNA49	China	*O. sativa ssp. indica*	19	91.61
DNA50	China	*O. sativa ssp. indica*	23	91.29
DNA66	Indonesia	*O. sativa ssp. indica*	27	92.17

Table S14 continued

Sample name	Origin	Cluster	Depth	Q30(%)
DNA88	Philippines	*O. sativa ssp. indica*	17	92.95
DNA9	India	*O. sativa ssp. indica*	24	91.99
DNA90	China	*O. sativa ssp. indica*	18	92.71
DNA91	China	*O. sativa ssp. indica*	19	92.95
DNA93	India	*O. sativa ssp. indica*	20	94.34
DNA94	China	*O. sativa ssp. indica*	17	92.12
DNA95	China	*O. sativa ssp. indica*	24	94.28
DNA96	India	*O. sativa ssp. indica*	33	94.21
S_81_75	China	*O. sativa ssp. indica*	21	96.71
S1_19_1	China	*O. sativa ssp. indica*	39	96.35
S10_10_3	China	*O. sativa ssp. indica*	32	96.69
S100	China	*O. sativa ssp. indica*	29	95.35
S10000	South Korea	*O. sativa ssp. indica*	13	93.26
S10001	China	*O. sativa ssp. indica*	35	94.99
S101	China	*O. sativa ssp. indica*	25	95.40
S10171	China	*O. sativa ssp. indica*	25	90.12
S10238	Philippines	*O. sativa ssp. indica*	45	96.00
S10385	Philippines	*O. sativa ssp. indica*	33	95.90
S104	China	*O. sativa ssp. indica*	29	95.78
S107	China	*O. sativa ssp. indica*	27	95.36
S10731	Nepal	*O. sativa ssp. indica*	11	91.19
S11_18_3	China	*O. sativa ssp. indica*	23	96.21
S110	China	*O. sativa ssp. indica*	30	95.62
S112	China	*O. sativa ssp. indica*	29	95.25
S11431	Philippines	*O. sativa ssp. indica*	36	95.38
S11446	India	*O. sativa ssp. indica*	34	95.53
S11624	Nepal	*O. sativa ssp. indica*	45	95.78
S11642	India	*O. sativa ssp. indica*	24	90.58
S11667	China	*O. sativa ssp. indica*	21	91.96
S11692	China	*O. sativa ssp. indica*	28	92.99
S11727	China	*O. sativa ssp. indica*	31	92.52
S11745	China	*O. sativa ssp. indica*	29	93.30
S11746	China	*O. sativa ssp. indica*	28	93.15
S11797	China	*O. sativa ssp. indica*	31	91.86
S11799	China	*O. sativa ssp. indica*	23	90.53
S11804	China	*O. sativa ssp. indica*	32	91.73
S11805	China	*O. sativa ssp. indica*	36	90.89
S11806	China	*O. sativa ssp. indica*	21	91.38
S11821	India	*O. sativa ssp. indica*	35	91.81
S11916	Sri Lanka	*O. sativa ssp. indica*	14	93.50
S11919	India	*O. sativa ssp. indica*	15	92.88
S11943	Nepal	*O. sativa ssp. indica*	11	94.45

Table S14 continued

Sample name	Origin	Cluster	Depth	Q30(%)
S11944	Nepal	*O. sativa ssp. indica*	10	94.24
S12135	Malaysia	*O. sativa ssp. indica*	15	92.45
S123	China	*O. sativa ssp. indica*	30	96.10
S132	China	*O. sativa ssp. indica*	29	95.99
S136	China	*O. sativa ssp. indica*	21	96.63
S137	China	*O. sativa ssp. indica*	26	95.89
S138	China	*O. sativa ssp. indica*	23	95.79
S146	China	*O. sativa ssp. indica*	30	95.05
S147	China	*O. sativa ssp. indica*	32	95.59
S15_17_5	China	*O. sativa ssp. indica*	29	95.72
S153	China	*O. sativa ssp. indica*	27	95.75
S155	China	*O. sativa ssp. indica*	32	95.70
S16_17_1	China	*O. sativa ssp. indica*	25	95.75
S163	China	*O. sativa ssp. indica*	29	95.40
S166	China	*O. sativa ssp. indica*	29	95.91
S171	China	*O. sativa ssp. indica*	27	95.56
S173	China	*O. sativa ssp. indica*	27	95.78
S187	China	*O. sativa ssp. indica*	26	96.00
S188	China	*O. sativa ssp. indica*	23	95.99
S189	China	*O. sativa ssp. indica*	25	94.17
S191	China	*O. sativa ssp. indica*	27	95.67
S2_10_2	China	*O. sativa ssp. indica*	23	96.26
S21_6_1	China	*O. sativa ssp. indica*	29	95.60
S23_1_7	China	*O. sativa ssp. indica*	28	95.75
S3_3_10	China	*O. sativa ssp. indica*	20	96.66
S30_19_2	China	*O. sativa ssp. indica*	22	95.87
S31_6_5	China	*O. sativa ssp. indica*	25	95.93
S32_10_1	China	*O. sativa ssp. indica*	29	95.69
S35_8_3	China	*O. sativa ssp. indica*	37	96.76
S37_2_5	China	*O. sativa ssp. indica*	23	96.05
S38_2_10	China	*O. sativa ssp. indica*	22	96.09
S434	Sri Lanka	*O. sativa ssp. indica*	24	95.20
S435	Philippines	*O. sativa ssp. indica*	24	95.16
S437	China	*O. sativa ssp. indica*	29	92.66
S438	China	*O. sativa ssp. indica*	30	92.85
S441	China	*O. sativa ssp. indica*	25	92.52
S443	China	*O. sativa ssp. indica*	24	95.31
S444	China	*O. sativa ssp. indica*	27	92.82
S445	China	*O. sativa ssp. indica*	28	95.37
S446	China	*O. sativa ssp. indica*	28	92.94
S452	China	*O. sativa ssp. indica*	27	92.09
S463	China	*O. sativa ssp. indica*	29	92.35

Table S14 continued

Sample name	Origin	Cluster	Depth	Q30(%)
S468	China	*O. sativa ssp. indica*	25	93.53
S469	China	*O. sativa ssp. indica*	24	93.17
S474	China	*O. sativa ssp. indica*	28	92.71
S475	China	*O. sativa ssp. indica*	25	95.67
S476	China	*O. sativa ssp. indica*	28	92.37
S48_15_1	Philippines	*O. sativa ssp. indica*	23	96.36
S483	China	*O. sativa ssp. indica*	30	95.35
S487	China	*O. sativa ssp. indica*	26	95.33
S488	China	*O. sativa ssp. indica*	32	94.99
S49_3_5	Philippines	*O. sativa ssp. indica*	23	96.20
S493	China	*O. sativa ssp. indica*	25	95.36
S494	China	*O. sativa ssp. indica*	27	95.60
S50_3_6	Philippines	*O. sativa ssp. indica*	23	96.31
S51_3_7	Philippines	*O. sativa ssp. indica*	24	96.27
S53_7	Philippines	*O. sativa ssp. indica*	22	96.54
S55_15_3	Philippines	*O. sativa ssp. indica*	22	96.74
S60_1_12	China	*O. sativa ssp. indica*	22	96.67
S62_15_6	China	*O. sativa ssp. indica*	27	96.60
S64_5_8	China	*O. sativa ssp. indica*	24	96.20
S66_17_6	China	*O. sativa ssp. indica*	20	96.82
S67_15_7	China	*O. sativa ssp. indica*	20	96.74
S68_73	China	*O. sativa ssp. indica*	22	96.82
S69_17_4	China	*O. sativa ssp. indica*	21	96.91
S80_74	China	*O. sativa ssp. indica*	23	96.42
S82_76	China	*O. sativa ssp. indica*	20	96.89
S83_17_7	China	*O. sativa ssp. indica*	22	96.74
S8392	Philippines	*O. sativa ssp. indica*	38	92.23
S86_18_6	China	*O. sativa ssp. indica*	24	96.39
S8645	China	*O. sativa ssp. indica*	37	94.36
S89_89	China	*O. sativa ssp. indica*	20	96.77
S8924	India	*O. sativa ssp. indica*	44	94.87
S8988	India	*O. sativa ssp. indica*	43	93.37
S9039	Sri Lanka	*O. sativa ssp. indica*	18	90.23
S91	China	*O. sativa ssp. indica*	30	96.51
S92	China	*O. sativa ssp. indica*	27	96.70
S9258	India	*O. sativa ssp. indica*	38	91.80
S93	China	*O. sativa ssp. indica*	21	96.81
S9324	China	*O. sativa ssp. indica*	50	96.06
S9547	India	*O. sativa ssp. indica*	36	93.35
S9605	India	*O. sativa ssp. indica*	44	95.26
S9611	India	*O. sativa ssp. indica*	43	96.38
S99	China	*O. sativa ssp. indica*	30	95.81

Table S14 continued

Sample name	Origin	Cluster	Depth	Q30(%)
S9922	SouthKorea	*O. sativa ssp. indica*	16	93.36
S9976	SouthKorea	*O. sativa ssp. indica*	11	92.67
195_S9917	Sri Lanka	*O. sativa ssp. indica*	24	95.64
319_I11621	India	*O. sativa ssp. indica*	27	93.55
356_B9696	Bangladesh	*O. sativa ssp. indica*	28	93.32
DNA100	China	*O. sativa ssp. japonica*	25	94.41
DNA53	China	*O. sativa ssp. japonica*	26	92.10
DNA55	Japan	*O. sativa ssp. japonica*	22	91.59
DNA59	Japan	*O. sativa ssp. japonica*	25	91.99
DNA61	Japan	*O. sativa ssp. japonica*	42	92.99
DNA67	North Korea	*O. sativa ssp. japonica*	22	91.63
DNA68	Japan	*O. sativa ssp. japonica*	21	92.91
DNA69	South Korea	*O. sativa ssp. japonica*	22	91.29
DNA70	South Korea	*O. sativa ssp. japonica*	20	91.95
DNA71	South Korea	*O. sativa ssp. japonica*	22	92.17
DNA82	Philippines	*O. sativa ssp. japonica*	17	92.88
DNA83	Philippines	*O. sativa ssp. japonica*	16	92.45
DNA86	India	*O. sativa ssp. japonica*	18	92.70
DNA89	China	*O. sativa ssp. japonica*	17	92.46
DNA97	China	*O. sativa ssp. japonica*	42	93.85
DNA99	China	*O. sativa ssp. japonica*	27	94.84
S10059	South Korea	*O. sativa ssp. japonica*	15	92.37
S10097	South Korea	*O. sativa ssp. japonica*	23	90.24
S109	China	*O. sativa ssp. japonica*	29	95.86
S114	China	*O. sativa ssp. japonica*	31	95.13
S115	China	*O. sativa ssp. japonica*	29	95.69
S116	China	*O. sativa ssp. japonica*	30	95.73
S11672	Nepal	*O. sativa ssp. japonica*	9	93.50
S118	China	*O. sativa ssp. japonica*	33	95.86
S11890	China	*O. sativa ssp. japonica*	12	91.98
S121	China	*O. sativa ssp. japonica*	35	95.87
S12217	South Korea	*O. sativa ssp. japonica*	14	93.95
S126	China	*O. sativa ssp. japonica*	30	95.68
S127	China	*O. sativa ssp. japonica*	30	95.73
S134	China	*O. sativa ssp. japonica*	31	96.04
S139	China	*O. sativa ssp. japonica*	32	97.02
S140	China	*O. sativa ssp. japonica*	30	94.39
S144	China	*O. sativa ssp. japonica*	35	95.85
S148	China	*O. sativa ssp. japonica*	31	96.25
S150	China	*O. sativa ssp. japonica*	29	95.07
S157	China	*O. sativa ssp. japonica*	32	95.17
S164	China	*O. sativa ssp. japonica*	32	95.87

Table S14 continued

Sample name	Origin	Cluster	Depth	Q30(%)
S175	China	*O. sativa ssp. japonica*	30	95.43
S176	China	*O. sativa ssp. japonica*	28	95.47
S177	Japan	*O. sativa ssp. japonica*	34	94.02
S178	China	*O. sativa ssp. japonica*	32	95.61
S180	China	*O. sativa ssp. japonica*	30	95.29
S183	China	*O. sativa ssp. japonica*	32	95.61
S185	China	*O. sativa ssp. japonica*	30	95.85
S192	China	*O. sativa ssp. japonica*	29	95.78
S439	China	*O. sativa ssp. japonica*	28	95.13
S442	Japan	*O. sativa ssp. japonica*	26	95.35
S451	China	*O. sativa ssp. japonica*	35	92.36
S453	China	*O. sativa ssp. japonica*	28	92.62
S454	China	*O. sativa ssp. japonica*	29	92.85
S457	China	*O. sativa ssp. japonica*	29	95.57
S459	China	*O. sativa ssp. japonica*	29	95.37
S461	China	*O. sativa ssp. japonica*	33	92.46
S462	China	*O. sativa ssp. japonica*	29	95.35
S464	China	*O. sativa ssp. japonica*	33	92.34
S482	China	*O. sativa ssp. japonica*	30	90.52
S484	China	*O. sativa ssp. japonica*	33	95.16
S490	China	*O. sativa ssp. japonica*	28	95.28
S491	China	*O. sativa ssp. japonica*	28	95.38
S54_21_4	China	*O. sativa ssp. japonica*	30	96.75
S7_28_1	China	*O. sativa ssp. japonica*	32	96.23
S76_71	Japan	*O. sativa ssp. japonica*	34	96.25
S9379	South Korea	*O. sativa ssp. japonica*	18	92.88
S97	China	*O. sativa ssp. japonica*	31	95.56
S9701	China	*O. sativa ssp. japonica*	16	92.59
S9702	China	*O. sativa ssp. japonica*	13	92.48
S9887	South Korea	*O. sativa ssp. japonica*	11	92.13
S9974	South Korea	*O. sativa ssp. japonica*	11	92.94
232_J10082	Japan	*O. sativa ssp. japonica*	23	95.88
291_J10079	Japan	*O. sativa ssp. japonica*	30	96.84
300_M9375	Malaysia	*O. sativa ssp. japonica*	34	92.91
DNA51	Philippines	*O. sativa ssp. japonica*	23	92.87
DNA52	Philippines	*O. sativa ssp. japonica*	20	91.90
DNA54	Philippines	*O. sativa ssp. japonica*	23	92.18
DNA57	Bhutan	*O. sativa ssp. japonica*	22	91.60
DNA58	Indonesia	*O. sativa ssp. japonica*	23	91.39
DNA60	Bhutan	*O. sativa ssp. japonica*	31	92.22
DNA63	Philippines	*O. sativa ssp. japonica*	25	92.56
DNA64	Bhutan	*O. sativa ssp. japonica*	22	92.17

Table S14 continued

Sample name	Origin	Cluster	Depth	Q30(%)
DNA65	Philippines	*O. sativa ssp. japonica*	22	92.33
DNA72	Philippines	*O. sativa ssp. japonica*	31	91.98
DNA73	South Korea	*O. sativa ssp. japonica*	31	91.72
DNA74	Japan	*O. sativa ssp. japonica*	18	93.19
DNA75	Japan	*O. sativa ssp. japonica*	19	94.08
DNA76	Japan	*O. sativa ssp. japonica*	18	92.42
DNA77	Japan	*O. sativa ssp. japonica*	17	92.75
DNA78	Japan	*O. sativa ssp. japonica*	24	93.14
DNA80	Japan	*O. sativa ssp. japonica*	20	93.53
DNA87	India	*O. sativa ssp. japonica*	18	92.35
S10485	Philippines	*O. sativa ssp. japonica*	13	93.56
S10656	Laos	*O. sativa ssp. japonica*	12	93.48
S10794	Indonesia	*O. sativa ssp. japonica*	13	92.23
S10799	Indonesia	*O. sativa ssp. japonica*	11	92.01
S10888	India	*O. sativa ssp. japonica*	14	92.24
S10895	India	*O. sativa ssp. japonica*	14	92.35
S10923	Thailand	*O. sativa ssp. japonica*	16	93.58
S10946	Indonesia	*O. sativa ssp. japonica*	12	92.97
S10949	Indonesia	*O. sativa ssp. japonica*	14	92.83
S11195	Myanmar	*O. sativa ssp. japonica*	8	93.84
S11328	Malaysia	*O. sativa ssp. japonica*	11	93.91
S11396	Indonesia	*O. sativa ssp. japonica*	13	91.23
S11900	Thailand	*O. sativa ssp. japonica*	23	90.61
S11922	Thailand	*O. sativa ssp. japonica*	13	93.40
S11924	Thailand	*O. sativa ssp. japonica*	56	92.17
S11929	Philippines	*O. sativa ssp. japonica*	20	94.28
S12071	Laos	*O. sativa ssp. japonica*	14	90.85
S12076	Laos	*O. sativa ssp. japonica*	11	90.07
S12129	Laos	*O. sativa ssp. japonica*	16	90.99
S12164	Cambodia	*O. sativa ssp. japonica*	15	92.24
S12200	Laos	*O. sativa ssp. japonica*	12	90.90
S12258	Laos	*O. sativa ssp. japonica*	14	93.98
S12262	Laos	*O. sativa ssp. japonica*	14	94.62
S12265	Laos	*O. sativa ssp. japonica*	14	90.54
S12266	Myanmar	*O. sativa ssp. japonica*	8	93.94
S12336	Laos	*O. sativa ssp. japonica*	11	93.68
S12349	Laos	*O. sativa ssp. japonica*	19	89.88
S12350	Laos	*O. sativa ssp. japonica*	14	94.04
S7902	Philippines	*O. sativa ssp. japonica*	21	95.01
S7909	Philippines	*O. sativa ssp. japonica*	18	95.03
S8010	Philippines	*O. sativa ssp. japonica*	18	94.75
S8011	Vietnam	*O. sativa ssp. japonica*	7	94.60

Table S14 continued

Sample name	Origin	Cluster	Depth	Q30(%)
S8279	Malaysia	*O. sativa ssp. japonica*	12	94.65
S8381	Malaysia	*O. sativa ssp. japonica*	15	94.71
S8436	Indonesia	*O. sativa ssp. japonica*	17	94.30
S8565	Thailand	*O. sativa ssp. japonica*	12	94.54
S8578	Vietnam	*O. sativa ssp. japonica*	11	94.30
S9301	Indonesia	*O. sativa ssp. japonica*	13	95.01
S9470	Indonesia	*O. sativa ssp. japonica*	18	94.95
S9995	SouthKorea	*O. sativa ssp. japonica*	11	91.17
229_I9083	India	*O. sativa ssp. japonica*	22	96.25
S11021	Pakistan	*O. sativa ssp. japonica*	12	94.03
S11258	India	*O. sativa ssp. japonica*	26	90.90
S11289	India	*O. sativa ssp. japonica*	22	90.19
S11625	Nepal	*O. sativa ssp. japonica*	38	93.45
S12094	Bangladesh	*O. sativa ssp. japonica*	18	93.22
S8326	India	*O. sativa ssp. japonica*	37	90.06
S8656	Pakistan	*O. sativa ssp. japonica*	43	93.81
S8813	India	*O. sativa ssp. japonica*	12	93.59
S11062	Bangladesh	*O. sativa ssp. japonica*	18	93.68
S11066	Bangladesh	*O. sativa ssp. japonica*	20	91.23

Table S15: The significant association sites based on GWAS data.

Traits	Chr	Start	End	P	Type	SV Length	Gene Numbers
GL	Chr02	5951743	5951743	0.000732622	insertion	24790	2
GL	Chr03	28471225	28471225	0.000367617	insertion	9719	1
GL	Chr04	11266553	11266553	0.000678044	insertion	23441	1
GL	Chr04	13706568	13706568	0.000893934	insertion	2601	3
GL	Chr05	25080470	25080470	0.000174249	insertion	226	2
GL	Chr05	4883232	4883232	0.000642552	insertion	236	0
GL	Chr05	27170453	27170453	0.0007689	insertion	621	0
GL	Chr06	27474890	27474890	0.000736691	insertion	429	0
GL	Chr07	24669321	24674315	0.000535013	insertion	4994	1
GL	Chr08	17406684	17406684	0.000311016	insertion	3764	3
GL	Chr08	11853865	11853865	0.000618143	insertion	1075	2
GL	Chr08	242574	242574	0.000892144	insertion	22809	3
GL	Chr08	17484450	17484450	0.000987216	insertion	2170	1
GL	Chr09	311539	311539	0.000354154	insertion	2873	1
GL	Chr11	11498043	11498043	0.000139807	insertion	11708	1
GL	Chr11	13650929	13650929	0.000156617	insertion	13166	2
GL	Chr11	13397460	13397460	0.000776423	insertion	2849	0
GL	Chr12	7891054	7891054	0.000272472	insertion	297	0
GL	Chr12	1225207	1225207	0.000496554	insertion	1051	0
GL	Chr12	1598818	1598818	0.000794604	insertion	260	0
GW	Chr01	36303339	36303339	0.000717469	insertion	5192	2
GW	Chr02	13312085	13312085	0.000410101	insertion	19143	4
GW	Chr02	25056990	25056990	0.000873719	insertion	11061	4
GW	Chr02	20180218	20180218	0.000986323	insertion	2014	1
GW	Chr03	33269226	33269226	0.000846249	insertion	5819	2
GW	Chr04	18570368	18570368	0.000375841	insertion	395	0
GW	Chr05	25426774	25426774	0.000505847	insertion	310	1
GW	Chr07	29162417	29162417	0.000576053	insertion	11170	1
GW	Chr08	5900739	5900739	0.000104251	insertion	532	1
GW	Chr09	6419665	6419665	6.39E-05	insertion	11311	4
GW	Chr10	20076395	20076395	0.000126798	insertion	565	1
GW	Chr10	11760366	11760366	0.000864492	insertion	21317	5
GW	Chr10	2426553	2426553	0.000907243	insertion	83	0
GW	Chr11	3469056	3469056	0.000505524	insertion	412	0
GW	Chr11	25217289	25217289	0.00066836	insertion	230	0
GW	Chr11	12952658	12952658	0.000914144	insertion	18586	3
LWR	Chr01	29750852	29750852	0.000340159	insertion	2469	0
LWR	Chr01	22693572	22693572	0.000649599	insertion	944	0
LWR	Chr01	11222061	11222061	0.000810224	insertion	1036	1

Table S15 continued

Traits	Chr	Start	End	P	Type	SV Length	Gene Numbers
LWR	Chr02	12781659	12781659	0.000280336	insertion	174	1
LWR	Chr02	20864751	20864751	0.000591255	insertion	250	0
LWR	Chr03	34578007	34578007	0.000833391	insertion	258	0
LWR	Chr04	6971913	6971913	0.000508954	insertion	2920	3
LWR	Chr04	35361713	35361713	0.000722657	insertion	3956	1
LWR	Chr05	4431493	4431493	0.000780804	insertion	1292	1
LWR	Chr05	23941706	23941706	0.000836609	insertion	101	0
LWR	Chr06	22509844	22509844	3.75E-05	insertion	11308	5
LWR	Chr07	7168661	7168661	1.07E-05	insertion	126	0
LWR	Chr07	15125931	15125931	0.000972577	insertion	134	0
LWR	Chr09	5967135	5967135	0.00016591	insertion	3119	2
LWR	Chr09	2421017	2421017	0.000392619	insertion	250	0
LWR	Chr09	6298121	6298121	0.000404743	insertion	2978	1
LWR	Chr09	2352655	2352655	0.000808235	insertion	424	0
LWR	Chr10	16909144	16909144	7.12E-05	insertion	7863	2
LWR	Chr10	4739965	4739965	8.56E-05	insertion	18444	5
LWR	Chr10	19105960	19105960	0.000100953	insertion	29226	3
LWR	Chr12	18567527	18567527	6.45E-05	insertion	953	0
LWR	Chr12	1475284	1475284	0.000362737	insertion	170	1
NB1	Chr01	90205	90205	0	insertion	87	0
NB1	Chr01	9789214	9789214	0	insertion	465	0
NB1	Chr01	15156177	15156177	0	insertion	3748	1
NB1	Chr01	15163208	15163208	0	insertion	1500	0
NB1	Chr01	25231269	25231373	0	deletion	104	0
NB1	Chr01	37753170	37753170	0	insertion	1181	0
NB1	Chr01	3092175	3092175	0.000529838	insertion	156	0
NB1	Chr02	8032541	8032541	0	insertion	814	0
NB1	Chr02	10983197	10983197	0	insertion	489	0
NB1	Chr02	11761061	11761061	0	insertion	491	0
NB1	Chr02	13231148	13231148	0	insertion	866	0
NB1	Chr02	33113555	33113555	0	insertion	976	1
NB1	Chr02	33280844	33280844	0	insertion	148	0
NB1	Chr02	8310442	8310442	0.000327223	insertion	346	0
NB1	Chr02	20889988	20889988	0.000538193	insertion	1954	1
NB1	Chr03	3002564	3002564	0	insertion	118	3
NB1	Chr03	8059017	8059017	0	insertion	513	1
NB1	Chr03	11935296	11935296	0	insertion	1587	1
NB1	Chr03	15179818	15179912	0	deletion	94	0
NB1	Chr03	17787898	17787898	0	insertion	349	0
NB1	Chr03	17793224	17793224	0	insertion	533	0
NB1	Chr03	19031034	19031034	0	insertion	511	0

Table S15 continued

Traits	Chr	Start	End	P	Type	SV Length	Gene Numbers
NB1	Chr03	21008402	21008402	0	insertion	163	0
NB1	Chr03	21491886	21491886	0	insertion	129	0
NB1	Chr03	23444749	23444749	0	insertion	104	0
NB1	Chr03	28253094	28253094	0	insertion	2004	0
NB1	Chr03	19859652	19859652	0.000302715	insertion	250	0
NB1	Chr04	6994550	6994550	0	insertion	1364	2
NB1	Chr04	18556467	18556467	0	insertion	605	0
NB1	Chr04	27773210	27773744	0	deletion	534	0
NB1	Chr04	33671742	33671965	0	deletion	223	0
NB1	Chr04	34429426	34429426	0	insertion	1466	4
NB1	Chr04	20024444	20024444	0.000480949	insertion	412	0
NB1	Chr05	5565772	5565772	0	insertion	123	0
NB1	Chr05	5712257	5712257	0	insertion	526	0
NB1	Chr05	9062230	9063118	0	deletion	888	0
NB1	Chr05	13448707	13448707	0	insertion	11769	6
NB1	Chr05	21600120	21600390	0	deletion	270	0
NB1	Chr05	23425710	23427505	0	deletion	1795	0
NB1	Chr05	24912345	24912345	0	insertion	2316	0
NB1	Chr05	25097574	25097574	0	insertion	608	3
NB1	Chr05	4356764	4356764	0.000140331	insertion	927	0
NB1	Chr05	13641839	13641839	0.000439308	insertion	12489	4
NB1	Chr05	4432235	4432235	0.000452993	insertion	1077	0
NB1	Chr06	1788350	1788350	0	insertion	2881	0
NB1	Chr06	6347792	6347792	0	insertion	3360	1
NB1	Chr06	6347797	6347797	0	insertion	603	0
NB1	Chr06	6384268	6384345	0	deletion	77	0
NB1	Chr06	20985698	20985698	0	insertion	1480	0
NB1	Chr06	28684262	28684314	0	deletion	52	0
NB1	Chr06	8804304	8804304	0.000104718	insertion	1378	1
NB1	Chr06	9586604	9586604	0.000568495	insertion	1130	0
NB1	Chr07	5332904	5332904	0	insertion	317	0
NB1	Chr07	6496849	6496849	0	insertion	302	0
NB1	Chr07	16596296	16596296	0	insertion	1183	1
NB1	Chr07	16612422	16612422	0	insertion	1650	6
NB1	Chr07	16724981	16724981	0	insertion	521	1
NB1	Chr07	18527021	18527021	0	insertion	560	0
NB1	Chr07	21508568	21508568	0	insertion	1584	1
NB1	Chr07	21557512	21559485	0	deletion	1973	0
NB1	Chr07	8323135	8323135	3.05E-05	insertion	104	0
NB1	Chr07	26550685	26550685	0.000121478	insertion	10703	1
NB1	Chr07	25220139	25220139	0.000616683	insertion	179	0

Table S15 continued

Traits	Chr	Start	End	P	Type	SV Length	Gene Numbers
NB1	Chr07	11893128	11893128	0.000857648	insertion	7770	1
NB1	Chr08	324805	324805	0	insertion	136	3
NB1	Chr08	27664774	27665866	0	deletion	1092	0
NB1	Chr08	19612193	19612193	0.000750793	insertion	576	0
NB1	Chr08	19167050	19167050	0.000769202	insertion	237	0
NB1	Chr09	16241159	16241519	0	deletion	360	0
NB1	Chr09	21517091	21517091	0	insertion	344	1
NB1	Chr09	22332786	22332786	0.000228302	insertion	152	1
NB1	Chr09	4069229	4069229	0.000300661	insertion	743	0
NB1	Chr09	20435198	20435198	0.000664971	insertion	5138	3
NB1	Chr10	1719647	1719647	0	insertion	125	0
NB1	Chr10	8254205	8254205	0	insertion	4384	3
NB1	Chr10	14127827	14127827	0	insertion	142	0
NB1	Chr10	14445556	14445556	0	insertion	3434	0
NB1	Chr10	22294027	22294139	0	deletion	112	0
NB1	Chr10	317911	317911	0.000332281	insertion	249	0
NB1	Chr11	2100242	2100242	0	insertion	129	0
NB1	Chr11	2282214	2282214	0	insertion	433	0
NB1	Chr11	24130706	24130706	0	insertion	538	0
NB1	Chr11	25255423	25255423	0	insertion	3327	3
NB1	Chr11	20535854	20535854	0.00066248	insertion	1610	1
NB1	Chr11	9378897	9378897	0.000990277	insertion	165	0
NB1	Chr12	5069478	5069478	0	insertion	470	0
NB1	Chr12	19145387	19145387	0.000247873	insertion	2376	1
NB2	Chr01	90205	90205	3.53E-07	insertion	83	0
NB2	Chr01	9789214	9789214	3.53E-07	insertion	465	0
NB2	Chr01	15156177	15156177	3.53E-07	insertion	3748	1
NB2	Chr01	15163208	15163208	3.53E-07	insertion	1500	0
NB2	Chr01	25231269	25231373	3.53E-07	deletion	104	0
NB2	Chr01	37753170	37753170	3.53E-07	insertion	1181	0
NB2	Chr01	27791908	27791908	9.72E-05	insertion	4125	2
NB2	Chr01	3394509	3394869	0.000557994	deletion	360	0
NB2	Chr01	31220025	31220025	0.000583063	insertion	266	1
NB2	Chr02	8032541	8032541	3.53E-07	insertion	814	0
NB2	Chr02	10983197	10983197	3.53E-07	insertion	489	0
NB2	Chr02	11761061	11761061	3.53E-07	insertion	491	0
NB2	Chr02	13231148	13231148	3.53E-07	insertion	866	0
NB2	Chr02	33113555	33113555	3.53E-07	insertion	976	1
NB2	Chr02	33280844	33280844	3.53E-07	insertion	107	0
NB2	Chr02	22399497	22399497	0.000173941	insertion	2380	1
NB2	Chr02	6935327	6935327	0.000329616	insertion	1468	1

Table S15 continued

Traits	Chr	Start	End	P	Type	SV Length	Gene Numbers
NB2	Chr02	23744085	23744085	0.000782147	insertion	810	1
NB2	Chr02	24740346	24740346	0.000980409	insertion	1508	0
NB2	Chr03	3002564	3002564	3.53E-07	insertion	142	3
NB2	Chr03	8059017	8059017	3.53E-07	insertion	513	1
NB2	Chr03	11935296	11935296	3.53E-07	insertion	1587	1
NB2	Chr03	15179818	15179912	3.53E-07	deletion	94	0
NB2	Chr03	17787898	17787898	3.53E-07	insertion	349	0
NB2	Chr03	17793224	17793224	3.53E-07	insertion	533	0
NB2	Chr03	19031034	19031034	3.53E-07	insertion	511	0
NB2	Chr03	21008402	21008402	3.53E-07	insertion	163	0
NB2	Chr03	21491886	21491886	3.53E-07	insertion	105	0
NB2	Chr03	23444749	23444749	3.53E-07	insertion	121	0
NB2	Chr03	28253094	28253094	3.53E-07	insertion	2004	0
NB2	Chr04	6994550	6994550	3.53E-07	insertion	1364	2
NB2	Chr04	18556467	18556467	3.53E-07	insertion	605	0
NB2	Chr04	27773210	27773744	3.53E-07	deletion	534	0
NB2	Chr04	33671742	33671965	3.53E-07	deletion	223	0
NB2	Chr04	34429426	34429426	3.53E-07	insertion	1466	4
NB2	Chr04	18798214	18798214	2.53E-05	insertion	370	2
NB2	Chr05	5565772	5565772	3.53E-07	insertion	90	0
NB2	Chr05	5712257	5712257	3.53E-07	insertion	526	0
NB2	Chr05	9062230	9063118	3.53E-07	deletion	888	0
NB2	Chr05	13448707	13448707	3.53E-07	insertion	11769	6
NB2	Chr05	21600120	21600390	3.53E-07	deletion	270	0
NB2	Chr05	23425710	23427505	3.53E-07	deletion	1795	0
NB2	Chr05	24912345	24912345	3.53E-07	insertion	2316	0
NB2	Chr05	25097574	25097574	3.53E-07	insertion	608	3
NB2	Chr05	22552935	22552935	0.000212443	insertion	727	1
NB2	Chr05	14026796	14026796	0.000744028	insertion	11827	3
NB2	Chr06	1788350	1788350	3.53E-07	insertion	2881	0
NB2	Chr06	6347792	6347792	3.53E-07	insertion	3360	1
NB2	Chr06	6347797	6347797	3.53E-07	insertion	603	0
NB2	Chr06	6384268	6384345	3.53E-07	deletion	77	0
NB2	Chr06	20985698	20985698	3.53E-07	insertion	1480	0
NB2	Chr06	28684262	28684314	3.53E-07	deletion	52	0
NB2	Chr06	22042003	22042003	5.33E-05	insertion	1216	1
NB2	Chr06	12549135	12549135	0.000452037	insertion	23516	7
NB2	Chr07	5332904	5332904	3.53E-07	insertion	317	0
NB2	Chr07	6496849	6496849	3.53E-07	insertion	302	0
NB2	Chr07	16596296	16596296	3.53E-07	insertion	1183	1
NB2	Chr07	16612422	16612422	3.53E-07	insertion	1650	6

Table S15 continued

Traits	Chr	Start	End	P	Type	SV Length	Gene Numbers
NB2	Chr07	16724981	16724981	3.53E-07	insertion	521	1
NB2	Chr07	18527021	18527021	3.53E-07	insertion	560	0
NB2	Chr07	21508568	21508568	3.53E-07	insertion	1584	1
NB2	Chr07	21557512	21559485	3.53E-07	deletion	1973	0
NB2	Chr07	22664042	22664042	0.000258186	insertion	209	0
NB2	Chr07	18621810	18621810	0.000533317	insertion	271	1
NB2	Chr07	11724183	11724183	0.000680115	insertion	10098	1
NB2	Chr08	324805	324805	3.53E-07	insertion	137	3
NB2	Chr08	27664774	27665866	3.53E-07	deletion	1092	0
NB2	Chr08	7708620	7708620	0.000303149	insertion	2008	2
NB2	Chr08	19566559	19566559	0.000438805	insertion	440	0
NB2	Chr08	6233521	6233521	0.000522216	insertion	1212	0
NB2	Chr08	26624795	26624895	0.000856551	deletion	100	0
NB2	Chr09	16241159	16241519	3.53E-07	deletion	360	0
NB2	Chr09	21517091	21517091	3.53E-07	insertion	344	1
NB2	Chr09	13501197	13501197	0.000129922	insertion	7444	2
NB2	Chr09	11875056	11875056	0.000865077	insertion	403	0
NB2	Chr10	1719647	1719647	3.53E-07	insertion	102	0
NB2	Chr10	8254205	8254205	3.53E-07	insertion	4384	3
NB2	Chr10	14127827	14127827	3.53E-07	insertion	107	0
NB2	Chr10	14445556	14445556	3.53E-07	insertion	3434	0
NB2	Chr10	22294027	22294139	3.53E-07	deletion	112	0
NB2	Chr10	22120879	22120879	0.000627626	insertion	243	0
NB2	Chr10	17588208	17588208	0.000933695	insertion	295	0
NB2	Chr11	2100242	2100242	3.53E-07	insertion	123	0
NB2	Chr11	2282214	2282214	3.53E-07	insertion	433	0
NB2	Chr11	24130706	24130706	3.53E-07	insertion	538	0
NB2	Chr11	25255423	25255423	3.53E-07	insertion	3327	3
NB2	Chr11	10102325	10102325	0.000497663	insertion	7975	4
NB2	Chr11	4405352	4405352	0.000753623	insertion	1147	1
NB2	Chr12	5069478	5069478	3.53E-07	insertion	470	0
NB2	Chr12	11833054	11833054	0.000493185	insertion	6501	1
NB2	Chr12	8860464	8860464	0.000776739	insertion	619	1
PH	Chr02	33160746	33160746	0.00037568	insertion	13772	2
PH	Chr02	16626625	16626625	0.000522282	insertion	4906	2
PH	Chr02	12781818	12781818	0.000534613	insertion	857	1
PH	Chr02	25695188	25695188	0.000740021	insertion	1120	1
PH	Chr03	10079206	10079206	0.00019185	insertion	128	0
PH	Chr03	35925300	35925300	0.00029495	insertion	685	2
PH	Chr03	16772500	16772500	0.000468633	insertion	258	1
PH	Chr03	22440021	22440021	0.000590291	insertion	350	0

Table S15 continued

Traits	Chr	Start	End	P	Type	SV Length	Gene Numbers
PH	Chr05	6349856	6349856	0.000433184	insertion	9955	2
PH	Chr05	7492728	7492728	0.000625014	insertion	2978	2
PH	Chr06	21612129	21612129	3.52E-05	insertion	6408	1
PH	Chr06	8679712	8679712	5.36E-05	insertion	1488	1
PH	Chr07	15777213	15777213	1.92E-05	insertion	277	0
PH	Chr07	20016522	20016762	0.000228218	deletion	240	0
PH	Chr07	16866102	16866102	0.000453339	insertion	35246	1
PH	Chr08	7250578	7250578	0.000334558	insertion	6913	1
PH	Chr08	6233521	6233521	0.000885939	insertion	1212	0
PH	Chr09	4459656	4459656	7.02E-05	insertion	1314	1
PH	Chr10	812092	812092	0.000510443	insertion	594	0
PH	Chr11	894479	894479	7.91E-05	insertion	226	0
PH	Chr11	19667645	19667645	0.000706874	insertion	2083	2
PH	Chr12	19140815	19140815	0.000801171	insertion	123	0
SBA	Chr01	90205	90205	0	insertion	110	0
SBA	Chr01	9789214	9789214	0	insertion	465	0
SBA	Chr01	15156177	15156177	0	insertion	3748	1
SBA	Chr01	15163208	15163208	0	insertion	1500	0
SBA	Chr01	25231269	25231373	0	deletion	104	0
SBA	Chr01	37753170	37753170	0	insertion	1181	0
SBA	Chr01	6480697	6480697	0.000311537	insertion	21223	4
SBA	Chr01	10724925	10724925	0.000358246	insertion	646	0
SBA	Chr01	30835231	30835231	0.000414755	insertion	1301	1
SBA	Chr02	8032541	8032541	0	insertion	814	0
SBA	Chr02	10983197	10983197	0	insertion	489	0
SBA	Chr02	11761061	11761061	0	insertion	491	0
SBA	Chr02	13231148	13231148	0	insertion	866	0
SBA	Chr02	33113555	33113555	0	insertion	976	1
SBA	Chr02	33280844	33280844	0	insertion	83	0
SBA	Chr02	22010910	22010910	0.000629997	insertion	249	0
SBA	Chr02	9954552	9954552	0.000791061	insertion	332	0
SBA	Chr02	21061297	21065829	0.000906025	deletion	4532	0
SBA	Chr03	3002564	3002564	0	insertion	125	3
SBA	Chr03	8059017	8059017	0	insertion	513	1
SBA	Chr03	11935296	11935296	0	insertion	1587	1
SBA	Chr03	15179818	15179912	0	deletion	94	0
SBA	Chr03	17787898	17787898	0	insertion	349	0
SBA	Chr03	17793224	17793224	0	insertion	533	0
SBA	Chr03	19031034	19031034	0	insertion	511	0
SBA	Chr03	21008402	21008402	0	insertion	163	0
SBA	Chr03	21491886	21491886	0	insertion	100	0

Table S15 continued

Traits	Chr	Start	End	P	Type	SV Length	Gene Numbers
SBA	Chr03	23444749	23444749	0	insertion	129	0
SBA	Chr03	28253094	28253094	0	insertion	2004	0
SBA	Chr04	6994550	6994550	0	insertion	1364	2
SBA	Chr04	18556467	18556467	0	insertion	605	0
SBA	Chr04	27773210	27773744	0	deletion	534	0
SBA	Chr04	33671742	33671965	0	deletion	223	0
SBA	Chr04	34429426	34429426	0	insertion	1466	4
SBA	Chr04	20577633	20577633	5.63E-05	insertion	226	0
SBA	Chr05	5565772	5565772	0	insertion	119	0
SBA	Chr05	5712257	5712257	0	insertion	526	0
SBA	Chr05	9062230	9063118	0	deletion	888	0
SBA	Chr05	13448707	13448707	0	insertion	11769	6
SBA	Chr05	21600120	21600390	0	deletion	270	0
SBA	Chr05	23425710	23427505	0	deletion	1795	0
SBA	Chr05	24912345	24912345	0	insertion	2316	0
SBA	Chr05	25097574	25097574	0	insertion	608	3
SBA	Chr05	18461260	18461531	0.000626417	deletion	271	0
SBA	Chr05	18218209	18218209	0.00070386	insertion	3872	2
SBA	Chr06	1788350	1788350	0	insertion	2881	0
SBA	Chr06	6347792	6347792	0	insertion	3360	1
SBA	Chr06	6347797	6347797	0	insertion	603	0
SBA	Chr06	6384268	6384345	0	deletion	77	0
SBA	Chr06	20985698	20985698	0	insertion	1480	0
SBA	Chr06	28684262	28684314	0	deletion	52	0
SBA	Chr06	29216859	29216859	0.000103022	insertion	690	0
SBA	Chr06	23654071	23654071	0.000910119	insertion	235	1
SBA	Chr07	5332904	5332904	0	insertion	317	0
SBA	Chr07	6496849	6496849	0	insertion	302	0
SBA	Chr07	16596296	16596296	0	insertion	1183	1
SBA	Chr07	16612422	16612422	0	insertion	1650	6
SBA	Chr07	16724981	16724981	0	insertion	521	1
SBA	Chr07	18527021	18527021	0	insertion	560	0
SBA	Chr07	21508568	21508568	0	insertion	1584	1
SBA	Chr07	21557512	21559485	0	deletion	1973	0
SBA	Chr08	324805	324805	0	insertion	95	3
SBA	Chr08	27664774	27665866	0	deletion	1092	0
SBA	Chr08	19123117	19123117	0.000519171	insertion	314	0
SBA	Chr09	16241159	16241519	0	deletion	360	0
SBA	Chr09	21517091	21517091	0	insertion	344	1
SBA	Chr09	22887840	22887840	5.95E-05	insertion	4460	2
SBA	Chr09	18839270	18839270	0.000377379	insertion	2879	2

Table S15 continued

Traits	Chr	Start	End	P	Type	SV Length	Gene Numbers
SBA	Chr09	2504993	2504993	0.000533134	insertion	2920	1
SBA	Chr09	6685459	6685459	0.000554396	insertion	23546	3
SBA	Chr10	1719647	1719647	0	insertion	111	0
SBA	Chr10	8254205	8254205	0	insertion	4384	3
SBA	Chr10	14127827	14127827	0	insertion	101	0
SBA	Chr10	14445556	14445556	0	insertion	3434	0
SBA	Chr10	22294027	22294139	0	deletion	112	0
SBA	Chr10	1010916	1010916	0.000496964	insertion	307	0
SBA	Chr10	12677730	12677730	0.000868482	insertion	11764	1
SBA	Chr10	14286862	14286862	0.000914045	insertion	516	0
SBA	Chr10	19506415	19506483	0.000948653	deletion	68	1
SBA	Chr11	2100242	2100242	0	insertion	80	0
SBA	Chr11	2282214	2282214	0	insertion	433	0
SBA	Chr11	24130706	24130706	0	insertion	538	0
SBA	Chr11	25255423	25255423	0	insertion	3327	3
SBA	Chr11	15563176	15563176	0.000243929	insertion	563	1
SBA	Chr12	5069478	5069478	0	insertion	470	0
SBA	Chr12	8860452	8860452	0.000428974	insertion	403	0
SBL	Chr01	34589188	34589188	0.000104293	insertion	13899	2
SBL	Chr01	25654202	25654202	0.000129947	insertion	3635	1
SBL	Chr02	35362821	35362821	1.36E-05	insertion	1893	0
SBL	Chr02	10634960	10634960	9.20E-05	insertion	4636	1
SBL	Chr02	2534701	2534701	0.00080927	insertion	10744	4
SBL	Chr03	28425838	28425838	0.000516187	insertion	14156	4
SBL	Chr03	25909589	25909589	0.000739403	insertion	205	0
SBL	Chr04	6727511	6727511	0.000183105	insertion	2809	3
SBL	Chr04	4772760	4772760	0.000377131	insertion	11157	5
SBL	Chr04	14409988	14409988	0.000656623	insertion	1224	0
SBL	Chr06	1192502	1192502	0.00017642	insertion	536	0
SBL	Chr06	19314706	19314706	0.000569276	insertion	2858	1
SBL	Chr07	5874874	5874874	0.000434088	insertion	3194	3
SBL	Chr08	10616828	10616828	0.000128329	insertion	631	0
SBL	Chr08	27554337	27554337	0.00027578	insertion	334	0
SBL	Chr08	5492309	5492309	0.000396818	insertion	6027	4
SBL	Chr08	11376554	11376554	0.000479301	insertion	15157	4
SBL	Chr09	6796069	6796311	0.000824338	deletion	242	0
SBL	Chr10	16244681	16244681	0.000633474	insertion	4904	2
SBL	Chr10	23404887	23404887	0.00084028	insertion	88	0
SBL	Chr11	18434057	18434057	0.000747955	insertion	16033	3
SBL	Chr12	1473553	1473553	4.72E-05	insertion	107	1
SBL	Chr12	929938	929938	0.000915431	insertion	89	0

Table S15 continued

Traits	Chr	Start	End	P	Type	SV Length	Gene Numbers
SBW	Chr01	3092004	3092004	0.000486434	insertion	2934	2
SBW	Chr01	4509460	4509460	0.00093505	insertion	1007	0
SBW	Chr02	11824550	11824550	0.000303808	insertion	264	0
SBW	Chr02	33113698	33113698	0.000332323	insertion	812	0
SBW	Chr02	3360869	3360869	0.0009572	insertion	891	0
SBW	Chr03	33446479	33446479	5.44E-05	insertion	12785	1
SBW	Chr04	34471453	34471453	0.000570355	insertion	5274	1
SBW	Chr05	5691577	5691577	8.42E-05	insertion	20456	2
SBW	Chr06	14785602	14785602	0.000131773	insertion	550	0
SBW	Chr07	5326054	5326054	0.00016033	insertion	4068	1
SBW	Chr07	20563854	20564555	0.000188962	deletion	701	0
SBW	Chr07	17701092	17701092	0.000459513	insertion	1325	0
SBW	Chr07	13222032	13222032	0.000472761	insertion	4140	0
SBW	Chr07	16596296	16596296	0.000669446	insertion	1183	1
SBW	Chr08	19315341	19315341	0.000997884	insertion	5047	2
SBW	Chr09	19648188	19648188	0.000216749	insertion	76	0
SBW	Chr09	7288848	7288848	0.000546499	insertion	11404	2
SBW	Chr09	7600916	7600916	0.000785967	insertion	874	0
SBW	Chr11	17523171	17523171	0.000380504	insertion	999	0
SBW	Chr11	22881621	22881621	0.000624335	insertion	21965	4
SBW	Chr11	9929882	9929882	0.000724527	insertion	3899	1
SBW	Chr11	8498768	8498768	0.000991642	insertion	2033	2
TGW	Chr01	30359146	30359146	0.000289748	insertion	386	1
TGW	Chr01	33401131	33401131	0.000413299	insertion	622	1
TGW	Chr02	3358154	3358154	0.000891928	insertion	356	1
TGW	Chr02	11831194	11831194	0.000939688	insertion	4252	1
TGW	Chr03	34365634	34365634	0.000737016	insertion	338	0
TGW	Chr03	29394172	29394172	0.000789113	insertion	400	0
TGW	Chr04	22546812	22546812	0.000260917	insertion	4987	2
TGW	Chr04	13408814	13408814	0.000389242	insertion	1040	0
TGW	Chr04	28849067	28849067	0.000717719	insertion	222	1
TGW	Chr04	1804066	1804066	0.000929693	insertion	9283	4
TGW	Chr06	30473825	30473825	3.34E-05	insertion	312	0
TGW	Chr06	24756851	24756851	0.000360067	insertion	7258	1
TGW	Chr06	5072965	5072965	0.000930056	insertion	689	0
TGW	Chr07	2183564	2183564	0.000405768	insertion	1116	0
TGW	Chr07	15303809	15303809	0.00055229	insertion	235	0
TGW	Chr07	16112548	16112548	0.000643229	insertion	1352	1
TGW	Chr08	19315341	19315341	0.000599841	insertion	5047	2
TGW	Chr08	16738863	16738863	0.000972389	insertion	160	0
TGW	Chr09	9860917	9860917	0.000254909	insertion	350	0

Table S15 continued

Traits	Chr	Start	End	P	Type	SV Length	Gene Numbers
TGW	Chr09	22266372	22266372	0.000615361	insertion	5684	4
TGW	Chr09	8841132	8841132	0.000701388	insertion	141	0
TGW	Chr10	14286065	14286065	0.000319231	insertion	1776	0
TGW	Chr12	7961921	7961921	0.000451055	insertion	731	0
TGW	Chr12	7866734	7866734	0.000849663	insertion	307	1

Table S16: 49 cloned genes overlapping with DELs (>10 kb) region.

Chromosome	Type	SV Length	MSU	gene region	Symbol	RAPdb
Chr10	deletion	22401	LOC_Os10g03660	exonic	OsADF	Os10g0125300
Chr10	deletion	11763	LOC_Os10g09850	exonic	OsDSR-1	Os10g0177200
Chr10	deletion	12959	LOC_Os10g31030	downstream	OsMTA4	Os10g0447600
Chr10	deletion	12181	LOC_Os10g31910	exonic	DEL1	Os10g0457200
Chr10	deletion	13221	LOC_Os10g33780	exonic	TAW1	Os10g0478000
Chr10	deletion	11309	LOC_Os10g33960	exonic	OSHB2	Os10g0480200
Chr10	deletion	13003	LOC_Os10g02584	exonic	OsGBP3	Os10g0115200
Chr10	deletion	57463	LOC_Os10g03660	exonic	OsADF	Os10g0125300
Chr10	deletion	34215	LOC_Os10g04720	exonic	Gnk2RLK-7	Os10g0136400
Chr10	deletion	14715	LOC_Os10g31030	exonic	OsMTA4	Os10g0447600
Chr10	deletion	19523	LOC_Os10g32920	exonic	HLL	Os10g0466700
Chr10	deletion	34215	LOC_Os10g04720	exonic	Gnk2RLK-7	Os10g0136400
Chr10	deletion	11402	LOC_Os10g33960	exonic	OSHB2	Os10g0480200
Chr10	deletion	20947	LOC_Os10g28200	exonic	OsGME1	Os10g0417600
Chr10	deletion	13029	LOC_Os10g36420	exonic	OsYABBY3|OsYAB4|TOB3	Os10g0508300
Chr10	deletion	15437	LOC_Os10g36924	exonic	OsNIP3;1|DTE1	Os10g0513200
Chr11	deletion	10178	LOC_Os11g40030	exonic	KRP3	Os11g0614800
Chr11	deletion	11996	LOC_Os11g30410	exonic	OsSAE1a	Os11g0497000
Chr11	deletion	10389	LOC_Os11g31330	intergenic	SS1|ONAC025	Os11g0512000
Chr11	deletion	20161	LOC_Os11g35390	exonic	OsMYB4P	Os11g0558200
Chr11	deletion	19815	LOC_Os11g43740	exonic	OsMADS68	Os11g0658700
Chr11	deletion	19819	LOC_Os11g44950	exonic	TIPS-11-9	Os11g0673200

Table S16 continued

Chromosome	Type	SV Length	MSU	gene region	Symbol	RAPdb
Chr11	deletion	13006	LOC_Os11g39670	exonic	TSCD11	Os11g0610900
Chr11	deletion	13450	LOC_Os11g36719	exonic	OsLOX10	Os11g0575600
Chr11	deletion	16342	LOC_Os11g10420	exonic	OsPI4K2	Os11g0209700
Chr11	deletion	12826	LOC_Os11g20310	exonic	Os11Gsk	Os11g0308100
Chr11	deletion	16731	LOC_Os11g39670	exonic	TSCD11	Os11g0610900
Chr11	deletion	10140	LOC_Os11g30410	exonic	OsSAE1a	Os11g0497000
Chr11	deletion	12851	LOC_Os11g09280	exonic	OsPDIL1;1	Os11g0199200
Chr11	deletion	55927	LOC_Os11g12810	exonic	OsSPS11	Os11g0236100
Chr11	deletion	11614	LOC_Os11g37990	exonic	OsCFM3	Os11g0592400
Chr11	deletion	13915	LOC_Os11g06780	exonic	OsRLCK311	Os11g0168600
Chr11	deletion	33070	LOC_Os11g11810	exonic	Os11gRGA5	Os11g0225300
Chr11	deletion	15016	LOC_Os11g35390	exonic	OsMYB4P	Os11g0558200
Chr11	deletion	15138	LOC_Os11g40610	exonic	OsELF4a	Os11g0621500
Chr12	deletion	15966	LOC_Os12g16160	exonic	OsCCT41	Os12g0262400
Chr12	deletion	10875	LOC_Os12g10184	exonic	PGL12	None
Chr12	deletion	19577	LOC_Os12g01370	exonic	OsFAD3	Os12g0104400
Chr12	deletion	50543	LOC_Os12g32650	exonic	OsCLT2	Os12g0511300
Chr12	deletion	13340	LOC_Os12g07590	exonic	OsPTP1	Os12g0174800
Chr12	deletion	12328	LOC_Os12g43600	exonic	Osgr-rbp4|Osgrp1	Os12g0632000
Chr12	deletion	10709	LOC_Os12g16160	exonic	OsCCT41	Os12g0262400
Chr12	deletion	52232	LOC_Os12g30520	exonic	OsPuf4	Os12g0488900
Chr12	deletion	15115	LOC_Os12g10540	exonic	OsMADS13	Os12g0207000
Chr12	deletion	17790	LOC_Os12g31810	exonic	OsCYCA2;1	Os12g0502300
Chr12	deletion	41874	LOC_Os12g33070	exonic	OsMYB46	Os12g0515300

Table S16 continued

Chromosome	Type	SV Length	MSU	gene region	Symbol	RAPdb
Chr12	deletion	10297	LOC_Os12g38180	exonic	OsHSP23.7	Os12g0569700
Chr12	deletion	11418	LOC_Os12g14150	exonic	OsKIX_11	Os12g0245100
Chr12	deletion	11448	LOC_Os12g21710	exonic	HTD12	Os12g0405200
